# Unique Mode of Cell Division by the Mycobacterial Genetic Resister Clones Emerging *De Novo* from the Antibiotic-Surviving Population

**DOI:** 10.1128/mSphere.00994-20

**Published:** 2020-11-18

**Authors:** Kishor Jakkala, Avraneel Paul, Atul Pradhan, Rashmi Ravindran Nair, Deepti Sharan, Sharmada Swaminath, Parthasarathi Ajitkumar

**Affiliations:** a Department of Microbiology and Cell Biology, Indian Institute of Science, Bangalore, Karnataka, India; Albert Einstein College of Medicine

**Keywords:** antibiotic resisters, hydroxyl radical, multinucleation, multiseptation, multiple constriction, multiple division, mycobacteria

## Abstract

The emergence of antibiotic genetic resisters of pathogenic bacteria poses a major public health challenge. The mechanism by which bacterial antibiotic genetic resister clones formed *de novo* multiply and establish a resister population remained unknown. Here, we delineated the unique mode of cell division of the antibiotic genetic resisters of Mycobacterium smegmatis and Mycobacterium tuberculosis formed *de novo* from the population surviving in the presence of bactericidal concentrations of rifampicin or moxifloxacin. The cells in the rifampicin/moxifloxacin-surviving population generated elevated levels of hydroxyl radical-inflicting mutations. The genetic mutants selected against rifampicin/moxifloxacin became multinucleated and multiseptated and developed multiple constrictions. These cells stochastically divided multiple times, producing sister-daughter cells phenomenally higher in number than what could be expected from their generation time. This caused an abrupt, unexpectedly high increase in the rifampicin/moxifloxacin resister colonies. This unique cell division behavior was not shown by the rifampicin resisters formed naturally in the actively growing cultures. We could detect such abrupt increases in the antibiotic resisters in others’ and our earlier data on the antibiotic-exposed laboratory/clinical M. tuberculosis strains, M. smegmatis and other bacteria in *in vitro* cultures, infected macrophages/animals, and tuberculosis patients. However, it went unnoticed/unreported in all those studies. This phenomenon occurring in diverse bacteria surviving against different antibiotics revealed the broad significance of the present study. We speculate that the antibiotic-resistant bacillary clones, which emerge in patients with diverse bacterial infections, might be using the same mechanism to establish an antibiotic resister population quickly in the continued presence of antibiotics.

**IMPORTANCE** The bacterial pathogens that are tolerant to antibiotics and survive in the continued presence of antibiotics have the chance to acquire genetically resistant mutations against the antibiotics and emerge *de novo* as antibiotic resisters. Once the antibiotic resister clone has emerged, often with compromise on growth characteristics, for the protection of the species, it is important to establish an antibiotic-resistant population quickly in the continued presence of the antibiotic. In this regard, the present study has unraveled multinucleation and multiseptation followed by multiple constrictions as the cellular processes used by the bacteria for quick multiplication to establish antibiotic-resistant populations. The study also points out the same phenomenon occurring in other bacterial systems investigated in our laboratory and others’ laboratories. Identification of these specific cellular events involved in quick multiplication offers additional cellular processes that can be targeted in combination with the existing antibiotics’ targets to preempt the emergence of antibiotic-resistant bacterial strains.

## Abstract (precis)

The bacterial pathogens that are tolerant to antibiotics and survive in the continued presence of antibiotics have the chance to acquire genetically resistant mutations against the antibiotics and emerge *de novo* as antibiotic resisters. Once the antibiotic resister clone has emerged, often with compromise on growth characteristics, for the protection of the species, it is important to establish an antibiotic-resistant population quickly in the continued presence of the antibiotic. In this regard, the present study has unraveled multinucleation and multiseptation followed by multiple constrictions as the cellular processes used by the bacteria for quick multiplication to establish antibiotic-resistant populations. The study also points out the same phenomenon occurring in other bacterial systems investigated in our laboratory and others’ laboratories. Identification of these specific cellular events involved in quick multiplication offers additional cellular processes that can be targeted in combination with the existing antibiotics’ targets to preempt the emergence of antibiotic-resistant bacterial strains.

## INTRODUCTION

Bacteria acquire genetic resistance using a variety of mechanisms (reviewed in references 1 and 2). Diverse genera of bacteria can get selected as genetically resistant strains against a wide range of both lethal and nonlethal concentrations of antibiotics (3). Bacteria, which survive under low or very low concentrations of antibiotics, do not undergo killing but acquire SOS-driven genetic mutations, emerge *de novo* as resistant mutants, proliferate, and get enriched (3). Minimum bactericidal concentration (MBC) of antibiotics or higher will kill most of the cells in the population. The small proportion of the cells that survive show a heterogeneous response to antibiotics, some of which are classical “persisters” that will grow and divide only when the antibiotic is withdrawn but will again stop growth and division upon reintroduction of the antibiotic, as termed decades ago (4) and redefined recently (5). Some persisters may grow and divide slowly, such as the isoniazid persisters of Mycobacterium smegmatis (6). The remaining cells in the population are also antibiotic tolerant and survive in the continued presence of MBC or lower concentrations of antibiotics using diverse molecular mechanisms (2, 7). A small proportion of the cells in the antibiotic-tolerant surviving population could acquire SOS-driven genetic mutations (8), grow and divide in the continued presence of the antibiotic, and establish an antibiotic-resistant genetically mutant population.

We recently reported the *de novo* emergence of rifampicin/moxifloxacin-resistant genetic mutants of Mycobacterium tuberculosis and Mycobacterium smegmatis from the respective antibiotic-surviving populations in the continued presence of bactericidal concentrations of antibiotics for prolonged duration *in vitro* (9–11). Besides genome-wide mutations, these antibiotic-selected genetic resisters carried hydroxyl radical-inflicted mutations at the rifampicin resistance-determining region (RRDR) in *rpoB* and the quinolone resistance-determining region (QRDR) in *gyrA*, respectively. The genetic resisters to the antibiotics regrew and divided to establish a mutant subpopulation in the continued presence of lethal concentrations of the antibiotics. Based on these observations of the antibiotic-exposed mycobacteria by us and similar observations made by other groups studying other bacteria (12–14), it was proposed that the antibiotic-surviving populations could be an evolutionary reservoir for antibiotic resisters (9–11, 15, 16).

Despite a fair knowledge of the molecular mechanisms of antibiotic resister generation, the physiological processes that drive the multiplication of the *de novo*-formed genetic resister clones from the antibiotic-surviving population remained unknown. Therefore, we designed the present study to find out the physiological mechanisms involved in the multiplication of genetic resister clones formed *de novo* against the antitubercular antibiotics rifampicin and moxifloxacin from the antibiotic-surviving populations of M. smegmatis and M. tuberculosis during prolonged exposure to different concentrations of these antibiotics.

## RESULTS

### Experimental rationale and strategy.

We exposed biological triplicates of M. smegmatis mid-log-phase (MLP) cultures to a minimum bactericidal concentration (MBC) of rifampicin and plated them on the antibiotic-free and antibiotic-containing (50× MBC of rifampicin) plates once every 6 h for 120 h for the determination of total CFU and the CFU of antibiotic resisters, respectively. We used 10× MBC rifampicin, in addition to 1× MBC rifampicin, for exposure because M. smegmatis, unlike M. tuberculosis, has ADP-ribosyl transferase (Arr) that inactivates rifampicin (17). We have confirmed that 10× MBC rifampicin inflicts lethality on M. smegmatis (10^8^ cells/ml) despite having Arr (11). Further, 10^6^ cells/ml were used for exposure to rifampicin to preempt the emergence of natural rifampicin resisters at ∼10^−8^ frequency (11, 18).

Based on the CFU on antibiotic-free plates at specific intervals of the exposure period, the killing phase with exponential reduction in the CFU, the antibiotic-surviving phase with no appreciable change in the CFU, and the regrowth phase with a steady rise in the CFU were temporally demarcated, as described earlier (10, 11). The cells from the antibiotic-surviving and the regrowing populations were analyzed for hydroxyl radical production using specific fluorescence dye, genetic mutations inflicted by hydroxyl radical by DNA sequencing, regrowth of the mutants using CFU determination on antibiotic plates, fluorescence and transmission electron microscopy and live-cell time-lapse imaging of cell division process, and determination of the expression levels of the genes involved in cell division, DNA replication and repair, SOS regulon, and so on.

In parallel, biological triplicates of M. smegmatis cultures were exposed to 10×, 3.75×, and 1× MBC of moxifloxacin (an antitubercular drug against gyrase) for 120 h, for the purpose of comparison of the physiological mechanisms. Here, 10^8^ cells/ml were used to preempt the natural resisters against moxifloxacin that arise at 10^−9^ frequency (10). However, plating of the moxifloxacin-exposed cells was performed only on moxifloxacin-free plates and not on moxifloxacin-containing plates. Also, the cells in the colonies were not sequenced for genetic mutations on the moxifloxacin-specific targets, *gyrA/gyrB*. This was because the *gyrA* mutations in the moxifloxacin resisters formed from the moxifloxacin-surviving cells were determined in our recent study on M. smegmatis cells surviving in the presence of 3.75× MBC of moxifloxacin (10).

In order to study the emergence of genetic resisters from the M. tuberculosis populations surviving in the continued presence of rifampicin/moxifloxacin, we took the data on the prolonged exposure of M. tuberculosis to rifampicin and moxifloxacin from our earlier published work (9). The reason to consider the M. tuberculosis data from the already published work was that the physiological events which we detected in the present study on M. smegmatis went unnoticed by us in the M. tuberculosis study and hence unreported (9). Nevertheless, irrespective of the antibiotics used, both the rifampicin/moxifloxacin-exposed M. tuberculosis and M. smegmatis MLP cultures gave similar triphasic responses to both the antibiotics. Therefore, the detailed study on the mechanism of emergence of antibiotic resisters from the antibiotic-surviving population was performed on the rifampicin/moxifloxacin-surviving populations of M. smegmatis only, and not on the antibiotic-exposed M. tuberculosis populations.

### The response of the M. smegmatis cultures exposed to 10× MBC rifampicin.

**FIG 1:**
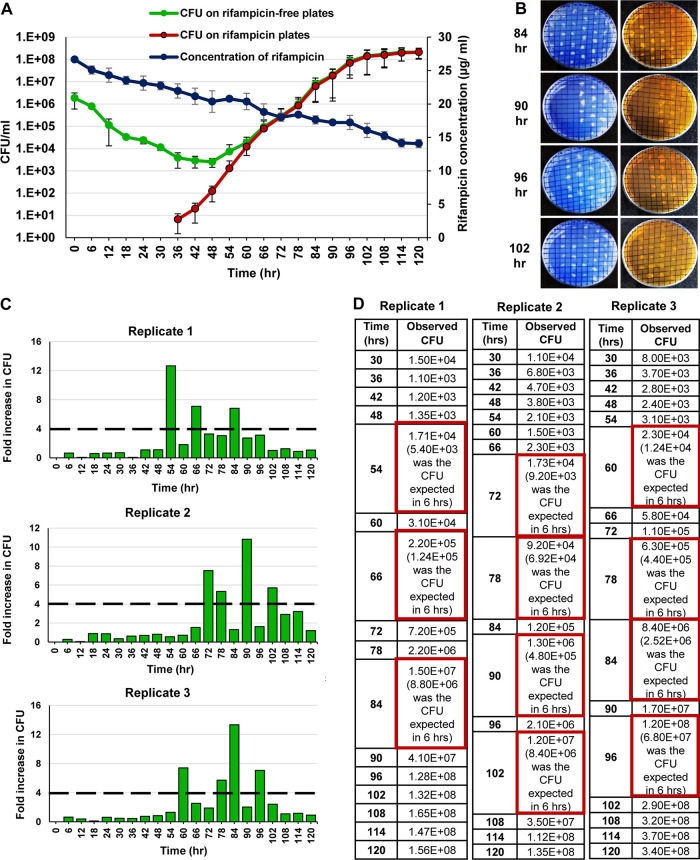
The CFU profile of M. smegmatis upon prolonged exposure to 10× MBC rifampicin. (A) CFU against 10× MBC rifampicin in culture, determined on rifampicin-free plates (green line). The killing phase (0 to 36 h), the rifampicin-surviving phase (36 to 48 h), and the regrowth phase (48 to 120 h). The CFU of the culture on 50× MBC rifampicin plates (red line). The rifampicin levels in the medium (blue line). *n* = 3. (B) Patching of colonies from the rifampicin-free master plate of the cultures from 84, 90, 96, and 102 h, grown in the presence of 10× MBC rifampicin, onto rifampicin-free plates (blue panels on the left) and rifampicin plates (50× MBC) (orange panels on the right). (C) The fold change in the CFU in 6-h periods. The dashed line indicates the expected 4-fold change in the CFU in 6 h. (D) The CFU values at 6-h intervals. The CFU spurts are shown in red boxes as the observed CFU and the expected CFU in parenthesis.

As inferred from the CFU on rifampicin-free plates, the 1× MBC rifampicin for 10^6^ cells/ml of M. smegmatis MLP culture was found to be 2.42 μg/ml (Fig. S1A). The CFU profile of the M. smegmatis MLP biological triplicate cultures (10^6^ cells/ml) exposed to 10× MBC rifampicin was determined from the CFU on the rifampicin-free plates. It showed a triphasic response involving, initially, a killing phase with an ∼3-log_10_ reduction in the CFU during 0 to 36 h (Fig. 1A, green line). The killing phase was followed by the antibiotic-surviving phase from 36 to 48 h, with no apparent change in the CFU, indicating the rifampicin-surviving population (Fig. 1A, green line). The rifampicin concentration was still ∼8 to 9× MBC (∼20 to 22 μg/ml) under which the cells were surviving (Fig. 1A, blue line). The ensuing regrowth phase, from 48 to 120 h, showed a steady rise of an ∼5-log_10_ increase in the CFU (Fig. 1A, green line). The regrowth occurred despite the high concentration of rifampicin, ranging from ∼6 to 8× MBC (∼15 to 20 μg/ml; Fig. 1A, blue line), indicating that this population probably contained rifampicin resisters (Fig. 1A, green line).

10.1128/mSphere.00994-20.1 FIG S1 (A) Determination of the minimum bactericidal concentration (MBC) of rifampicin for 10^6^ cells/ml of M. smegmatis. The MBC of rifampicin for 10^6^ cells/ml of M. smegmatis was determined to be 2.42 μg/ml (*n* = 4). (B) HPF fluorescence profile of the cells in the late rifampicin-surviving phase followed by the regrowth phase. The reciprocal relationship between the CFU and HPF fluorescence. The fluorescence levels were normalized with CFU. The readings were taken on a Tecan multiwell plate reader. (C to E) Mutations in the RRDR of *rpoB*. (C and D) Comparison of the RRDR sequence of the wild-type genome with the RRDR sequence of the cells from the (C) rifampicin plates and (D) rifampicin-free plates. In both panels C and D, *n* = 3 experimental samples, each with biological triplicates for the sequence determination. (E) RRDR mutations in the six rifampicin-resistant mutants selected from the MLP culture. Download FIG S1, TIF file, 2.0 MB. Copyright © 2020 Jakkala et al. 2020 Jakkala et al. This content is distributed under the terms of the Creative Commons Attribution 4.0 International license.

As inferred from the CFU on rifampicin plates, the CFU on the 50× MBC rifampicin plates started appearing from 36 h onward and increasing all the way up to 120 h, hinting at the emergence of rifampicin resisters (Fig. 1A, red line). The extrapolation of the slope of the resister generation graph (Fig. 1A, red line) to the *x* axis showed that the resister clones emerged during the treatment and were not present in the initial periods of the culture. Further, the resister clones seemed to have emerged before the antibiotic-surviving period from where the multinucleated cells began to be formed. One may note that the slope of the red line in Fig. 1A was consistent with logarithmic growth of the resistant population in the presence of the antibiotic. From the 60th h of exposure onward, the CFU on rifampicin plates overlapped with the CFU on the rifampicin-free plates, indicating that every CFU that emerged from 60 h onward might have been from a rifampicin resister cell (Fig. 1A, red line). This premise was confirmed when every single colony which was in the rifampicin-free master plate of the cultures from the 84 h, 90 h, 96 h, and 102 h of the regrowth phase grew on both rifampicin-free and 50× MBC rifampicin plates, when patch-plated (Fig. 1B).

### Stochastic high spurts in the cell number in the regrowing population.

**FIG 2:**
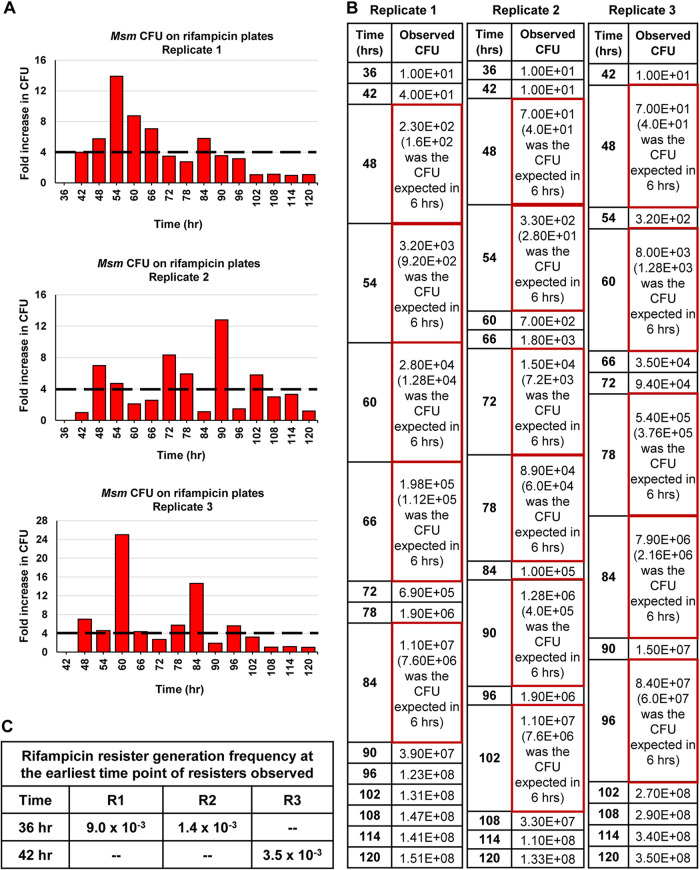
CFU spurts of M. smegmatis rifampicin resisters against 10× MBC rifampicin. (A) The fold change in the CFU compared to the CFU of the previous time point of the 6-h time intervals for the biological triplicates. The dashed line indicates the maximum expected 4-fold change in the CFU in 6-h periods. (B) The observed CFU values of the biological triplicates and the expected 4-fold CFU values in parenthesis. The observed CFU spurts are shown in red boxes. (C) Rifampicin resister generation frequency of the cells at the earliest time point of resister emergence in the three replicate cultures. *n* = 3.

Since the mass doubling time of M. smegmatis cells in the Middlebrook 7H9 medium is ∼3 h (19), a 2-fold increase in the CFU was expected in 3 h and 4-fold in 6 h. However, to our surprise, in all the three replicates, ∼8 to 13-fold higher stochastic spurts in the cell number, instead of the expected 4-fold, were indicated by the CFU on the rifampicin-free plates during one or the other 6-h periods, starting from the 48 to 54 h period, in the regrowth phase (Fig. 1C and D; *n* = 3). The CFU values of the 6-h aliquots, from the antibiotic-surviving and regrowth phase populations, on the 50× MBC rifampicin plates also revealed ∼8 to 25-fold higher stochastic spurts in cell number, instead of the expected 4-fold increase, shown by the CFU readout during one or the other 6-h periods among the biological triplicates (Fig. 2A and B; *n* = 3).

### High rifampicin resister generation frequency.

The rifampicin resister generation frequency of the cells of the 36-h time point, which is the first time point of appearance of resisters in two of the biological replicates, were 9 × 10^−3^ and 1.4 × 10^−3^ (Fig. 2C). The third replicate showed resister emergence at 42 h with a resister generation frequency of 3.5 × 10^−3^. The rifampicin resister generation frequency of ∼10^−3^ was ∼5-log_10_-fold higher than the natural resister generation frequency of 10^−8^ of M. smegmatis against rifampicin (11, 18). The high resister generation frequency alluded to elevated levels of the DNA-nonspecific mutagen, hydroxyl radical (20–22), as found by us in the rifampicin/moxifloxacin-exposed M. tuberculosis and M. smegmatis antibiotic-surviving cells (9–11) and in the rifampin exposed M. tuberculosis cells (23) and by others in other bacterial systems exposed to antibiotics (12–14).

### Elevated levels of hydroxyl radical in the rifampicin-surviving cells.

High levels of hydroxyl radical-specific hydroxyphenyl fluorescein (HPF) fluorescence could be detected when monitored from 48 h onward, which is the late stage of the rifampicin-surviving phase (Fig. S1B; see Fig. 1A). The HPF fluorescence was steady until 54 h (beginning of the regrowth phase) and then gradually declined until 90 h. Reciprocally, the CFU began to rise steadily after 54 h with the emergence of rifampicin resisters in the regrowth phase and reached a plateau at 90 h (Fig. S1B). It indicated that the generation of hydroxyl radical was at its highest in the antibiotic-surviving population, and then it declined when the cells in the rifampicin-surviving population started regrowth and division, probably after they had gained rifampicin-resistant mutations and thereby became free from the antibiotic stress.

### Regrowing cells were rifampicin-resisters with mutation at RRDR in *rpoB*.

Consistent with hydroxyl radical being a sequence-nonspecific DNA mutagen (20–22), the 96-h colonies from the rifampicin plates showed mutations at the RRDR of *rpoB* in all the colonies (Fig. S1C). Sequencing of the colonies from the rifampicin-free plate, obtained from the 96-h culture growing in the presence of 50× MBC of rifampicin, also showed mutation in the RRDR of *rpoB* (Fig. S1D). The nucleotide changes, C→T and A→G, were indicative of mutations caused by high levels of hydroxyl radical, as reported (20–22, 24). It may be recalled that every single colony from the 96-h culture, when patch-plated from the rifampicin-free master plate into both rifampicin-free and 50× MBC rifampicin plate, grew and formed colonies (see Fig. 1B). These observations confirmed that the mutations were already present in the cells regrowing from the rifampicin-surviving population in the rifampicin-containing liquid culture itself and were not generated on the selection plate containing rifampicin.

### Naturally formed rifampicin resisters do not show cell number spurts

Since the cell number spurts were found among the rifampicin-surviving population and rifampicin-resistant mutants that emerged from the population surviving against high MBC of rifampicin, we verified whether this phenomenon would be shown by the natural rifampicin resisters emerging from the MLP cultures. For this purpose, we used the rifampicin resisters containing the RRDR mutation that were selected by plating the MLP culture on 50× MBC rifampicin plates (Fig. S1E). These mutants, when cultured in 10× MBC rifampin-containing liquid medium and plated on antibiotic-free plate, showed only the expected 2-fold increase in the CFU within ∼3 h of generation time (Fig. S2A and B). Thus, the abnormally high spurt in the cell number was a unique cell division behavior of the population surviving in the continued presence of 10× MBC rifampicin and of the rifampicin resisters that emerged from the antibiotic-surviving population, and not that of the naturally formed rifampicin resisters in the MLP population.

10.1128/mSphere.00994-20.2 FIG S2 (A and B) Growth, division, and fold change in the CFU of the natural rifampicin resisters selected from the MLP culture. (A) Growth curve of one of the six M. smegmatis rifampicin-resistant mutants in the presence of 10× MBC rifampicin. (B) The fold change in the CFU of the rifampicin-resistant mutant cultured in panel A. The normal 2-fold change in the CFU expected in 3-h intervals is indicated by the dotted line. *n* = 3. (C to E) Growth curve, fold change in the CFU, and rifampicin resister generation frequency of M. smegmatis MLP rifampicin-unexposed cultures. (C) Growth curve of MLP-untreated cells (10^6^ cells/ml) obtained from rifampicin-free and rifampicin-containing plates. (D) Fold change in the CFU of the cultures at specific time intervals. (E) Rifampicin resister generation frequency. (*n* = 3). (F and G) Estimation of glycerol levels in the M. smegmatis MLP untreated cultures. (F) Standard curve for glycerol estimation with different concentrations of glycerol. (G) Estimation of glycerol levels in the spent medium of the MLP-untreated cultures (*n* = 3). Download FIG S2, TIF file, 0.4 MB. Copyright © 2020 Jakkala et al. 2020 Jakkala et al. This content is distributed under the terms of the Creative Commons Attribution 4.0 International license.

### Cell number spurts were shown by the rifampicin-exposed cultures only.

At this juncture, it may be noted that the high spurts in cell number, as observed from the CFU on the rifampicin-free plates and rifampicin-containing plates, were shown by the rifampicin-surviving cells at 48 h of the culture (late rifampicin-surviving phase) and by the cells regrowing from the rifampicin-surviving population (from 54 h of exposure onward) (see Fig. 1D and 2B). Therefore, it was possible that M. smegmatis cultures grown for an equivalent long duration in the absence of rifampicin also might show cell number spurts, probably due to the likely nutritional stress. In order to verify this possibility, we cultured biological triplicates of MLP cells for 120 h in the absence of rifampicin, like in the case of rifampicin-exposed cultures. Equal aliquots from these cultures were plated on rifampicin-free and rifampicin-containing (50× MBC) plates. The triplicate cultures did not show any spurt in cell number throughout the prolonged exposure for 120 h, as revealed by the CFU (Fig. S2C and D). The rifampicin resister generation frequency of these cultures was only 10^−8^, which is the natural rifampicin resister generation frequency of the M. smegmatis wild-type (WT) strain exposed to rifampicin (Fig. S2E) (11, 18).

The glycerol levels in the culture medium, as estimated from the culture supernatants from the respective time points of plating, had decreased to nil by 24 h (Fig. S2F and G). Thus, the cells did not show spurts in cell number or increase in the rifampicin resister generation frequency even though they suffered from nutritional stress. In contrast, the glycerol levels in the 10× MBC rifampicin-containing culture declined only to a negligible extent up to 66 h of exposure, continuing to remain steady until 102 h of exposure, and declining steeply thereafter (Fig. S3A and B). The steep decline in the glycerol levels correlated with the regrowth of the cells in the culture (Fig. S3C; correlate with Fig. S3B). These experiments showed that the cells showed cell number spurts only in response to antibiotic stress imposed by the continued presence of the antibiotic, even though they were not suffering from nutritional stress.

10.1128/mSphere.00994-20.3 FIG S3 (A to C) Estimation of glycerol in the 10× MBC rifampicin-containing M. smegmatis cultures during the regrowth phase. (A) Standard curve for glycerol estimation with different concentrations of glycerol. (B) The concentration of glycerol present in the culture at different time points during the exposure of the cells to rifampin. (C) Growth curve of the cells measured by observing the cell density at an optical density at 600 nm (OD_600_) at different time points. *n* = 3. (D to N) Transmission electron micrographs of the M. smegmatis cells, regrowing from the rifampicin-surviving population at 96 h. Cells with (D to K) multiple nucleoids, (G to J) multiple septa, (F, G, I, and J) polar septum with nucleoids, and (J and K) anucleated portions due to polar septation. (L to N) Mid/polar septation in shorter-sized cells (∼1 μm in length). *n* = 1,081 cells for panels D to K. Arrows indicate nucleoids/septa/anucleated cells. (O) Genomic DNA profile obtained from the cells from six different colonies taken from the 96-h sample, showing the intact genome. Download FIG S3, TIF file, 2.4 MB. Copyright © 2020 Jakkala et al. 2020 Jakkala et al. This content is distributed under the terms of the Creative Commons Attribution 4.0 International license.

### Multinucleated elongated cells in the regrowing population.

We hypothesized that the cell number spurts during the regrowth phase could be due to three possibilities: (i) the formation of multiple nucleoids and multiple septa in the mother cells followed by their multiple constrictions to generate multiple sister-daughter cells, as found in the filamentous temperature-sensitive (*fts*) mutants of Escherichia coli when shifted from nonpermissive to permissive temperature (25), (ii) multinucleated mother cells undergoing division asymmetrically from the tips of elongated cells, as found in the early stationary-phase M. tuberculosis cultures (26) and ciprofloxacin-treated filamentous E. coli cells (27), and/or (iii) mononucleated cells undergoing growth and division with shorter generation time as found in the cells having various mass doubling time from 34 min to 123 min in E. coli cultures (28).

**FIG 3:**
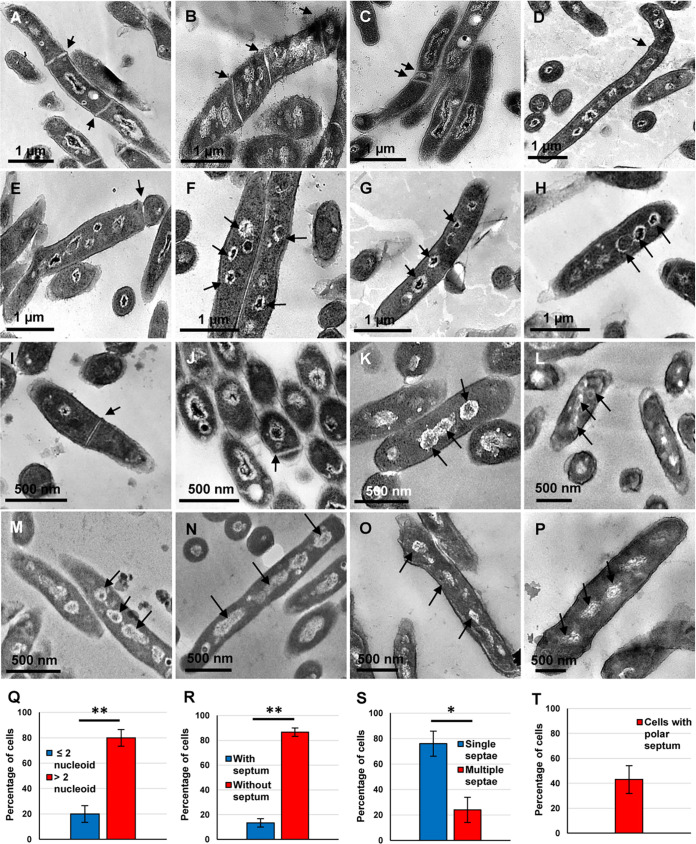
Transmission electron micrographs and quantitation of the M. smegmatis cells, regrowing from the rifampicin-surviving population, taken at different time points during rifampicin exposure. (A to H) Elongated cells taken at 96 h. (I and J) Shorter-sized (∼1 μm in length) cells at 96 h. (K to P) Regrowing cells taken at (K) 60 h, (L) 66 h, (M) 72 h, (N) 78 h, (O) 84 h, and (P) 90 h. (Q to T) Quantitation of the cells with nucleoids/septa from 96-h samples. Arrow heads indicate nucleoids/septa. *n* = 1,081 cells for panels Q and R, and *n* = 210 septated cells for panels S and T. Statistical significance was calculated using two-tailed paired *t* test; *, *P* ≤ 0.05; **, *P ≤ *0.01.

Among the three possibilities, the presence of elongated cells with multiple condensed or spread out nucleoids was confirmed by the transmission electron micrographs (TEM) of the cells aliquoted from the cultures at every 6 h during the regrowth phase from 60 h to 96 h (Fig. 3A to P; more images in Fig. S3D to N). The TEM images of the cells at 96 h (regrowth phase) showed that 79.95 ± 6.55% of the cells had >2n nucleoid content, while the remaining 20.05 ± 6.55% possessed ≤2n nucleoid content (*n* = 1,081) (Fig. 3Q). The elongated nucleoids (Fig. 3C, M, and N) were reminiscent of the morphology of the replicating and segregating nucleoids during cell division in mycobacteria (see Fig. 2a, b, and e in reference 29). The genomic DNA profile obtained from the cells from six different colonies taken from the 96-h sample, showed intact genome, thereby confirming that the multiple nucleoids in the elongated cells were not fragmented/degraded nucleoids/genomes (Fig. S3O).

**FIG 4:**
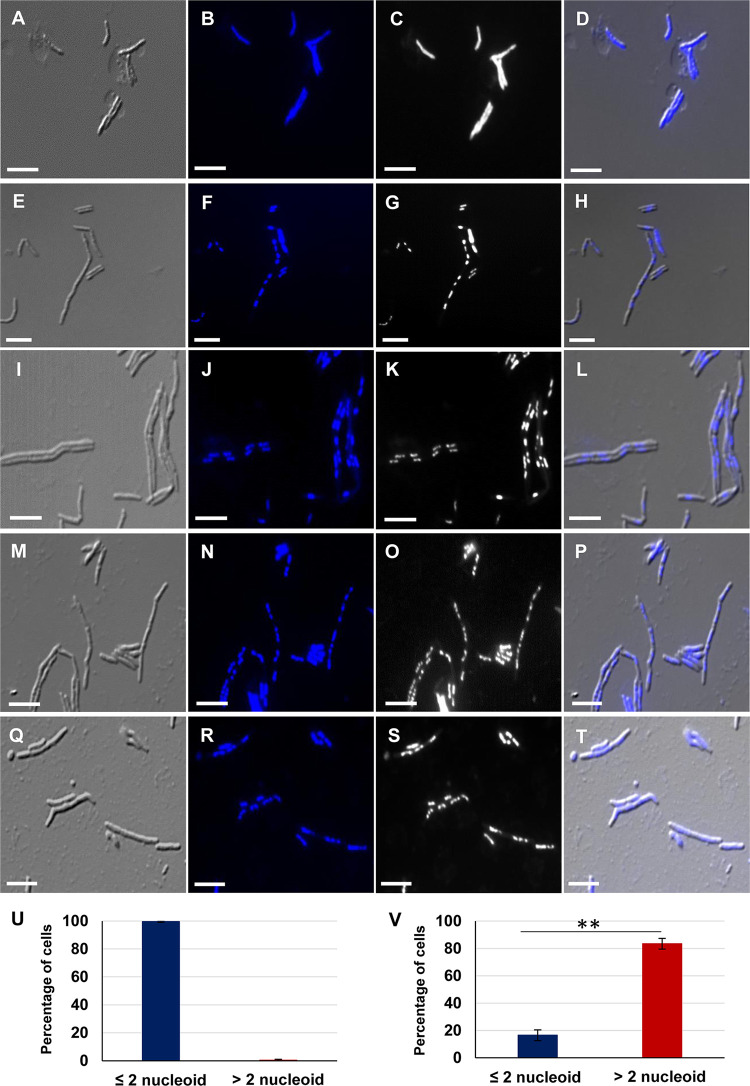
Hoechst 33342 staining of M. smegmatis cells regrowing from the rifampicin-surviving population and MLP. (A to D) MLP cells prior to rifampicin exposure (0 h). (E to T) Cells (96 h) regrowing from the rifampicin-surviving population during antibiotic exposure. DIC images of (A) MLP cells and (E, I, M, and Q) regrowing cells. The Hoechst fluorescence of the nucleoid profile of (B) MLP cells and (F, J, N, and R) regrowing cells. The fluorescence profile of the nucleoid in the gray scale of: (C) MLP cells and (G, K, O, and S) regrowing cells. The DIC-Hoechst 33342 fluorescence merged images of (D) MLP cells and (H, L, P, and T) regrowing cells. (U and V) Quantitation of the cells: (U) prior to rifampicin exposure (*n* = 183 from biological triplicates). (V) 96 h of rifampicin exposure (*n* = 896 cells from biological triplicates). Scale bar = 5 μm. Results were analyzed using two-tailed paired *t* test; **, *P* ≤ 0.01.

The staining of the nucleoids of the cells from the MLP samples with the DNA-specific dye, Hoechst 33342, showed that 99.60 ± 0.61% of the MLP cells at 0 h (i.e., before antibiotic exposure) possessed ≤2n nucleoid content (*n* = 183 from biological triplicates) (Fig. 4A to D and U). In contrast, 83.48 ± 3.91% cells (79.95 ± 6.55% as per TEM data) from the 96-h regrowth phase possessed >2n nucleoid content, with only 16.51 ± 3.91% cells (20.05 ± 6.55% as per TEM data) having ≤2n nucleoid content (*n* = 896 cells from biological triplicates) (Fig. 4E to T and V). The quantitation of the nucleoid stained cells from the 96-h samples correlated with the quantitation of nucleoids from the TEM data of 96-h samples (see Fig. 3A to P; Fig. S3D to N), both showing the presence of high proportions of the cells with multiple nucleoids in the subpopulation regrowing from the rifampicin-surviving population.

### Multiple septa in multinucleated elongated cells.

The TEM images of the cells regrowing from the rifampicin-surviving population, at every 6-h during the regrowing phase from 60 h to 96 h, showed multiple septa as well (Fig. 3A and B; more images in Fig. S3G to J). Short-sized cells (∼0.5 μm to 1 μm) with a mid/polar septum also could be observed (Fig. 3I and J; more images in Fig. S3L to N). Among the cells at 96 h, 13.47 ± 3.41% of the cells were septated, and the remaining 86.53 ± 3.41% did not possess a septum (*n* = 1,081) (Fig. 3R). Further, among the total number of septated cells at 96 h, 23.97 ± 9.88% possessed multiple septa, while 76.03 ± 9.88% of cells contained a single septum (*n* = 210) (Fig. 3S). Among the septated cells (*n* = 210), 43.09 ± 11.14% of the cells had a polar septum showing anucleated portions of the cells (Fig. 3E, J, and T; more images in Fig. S3G, I to K, and N). The atomic force micrographs (AFM) of the 96-h cells regrowing from the rifampicin-surviving population revealed the presence of multiple ridges (Fig. S4A to C). These were indicative of multiple septa beneath, for potential multiple constrictions, as predicted in an earlier study using scanning electron microscopy (SEM; 26) and in a recent study using AFM (29).

10.1128/mSphere.00994-20.4 FIG S4 (A to C) Atomic force micrographs of rifampicin-exposed M. smegmatis cells. (A) Three-dimensional (3D) representative image of a cell taken from the 96-h regrowth phase showing a ridge-and-trough type of cell surface. White arrows indicate circular ridges, probably corresponding to multiple septa beneath, and green arrows indicate troughs. (B) The flattened image indicates the area selected for making the line profiles (red and green lines). (C) Line profiles (red and green lines) representing the (red) smooth surface of MLP cells and (green) ridge-and-trough type of cell surface of the cell regrowing from the rifampicin-surviving population. (D and E) High CFU spurts of rifampicin resisters from the M. smegmatis cells regrowing from the rifampicin-surviving population during prolonged exposure to 1× MBC rifampicin. (D) The fold-change in CFU compared to the CFU of the previous time point of the biological triplicates on 50× MBC rifampicin plates. The dotted line indicates the expected 4-fold increase in the CFU in 6 h. (*n* = 3). (E) The actual CFU values of the biological triplicates. The CFU spurts in 6 h are indicated in red boxes with the expected 4-fold CFU in parenthesis. (F) The rifampicin resister generation frequency calculated from the earliest time point of resister emergence. (*n* = 3). Download FIG S4, TIF file, 1.7 MB. Copyright © 2020 Jakkala et al. 2020 Jakkala et al. This content is distributed under the terms of the Creative Commons Attribution 4.0 International license.

### Time-lapse live-cell imaging confirmed multiple constriction/division.

**FIG 5:**
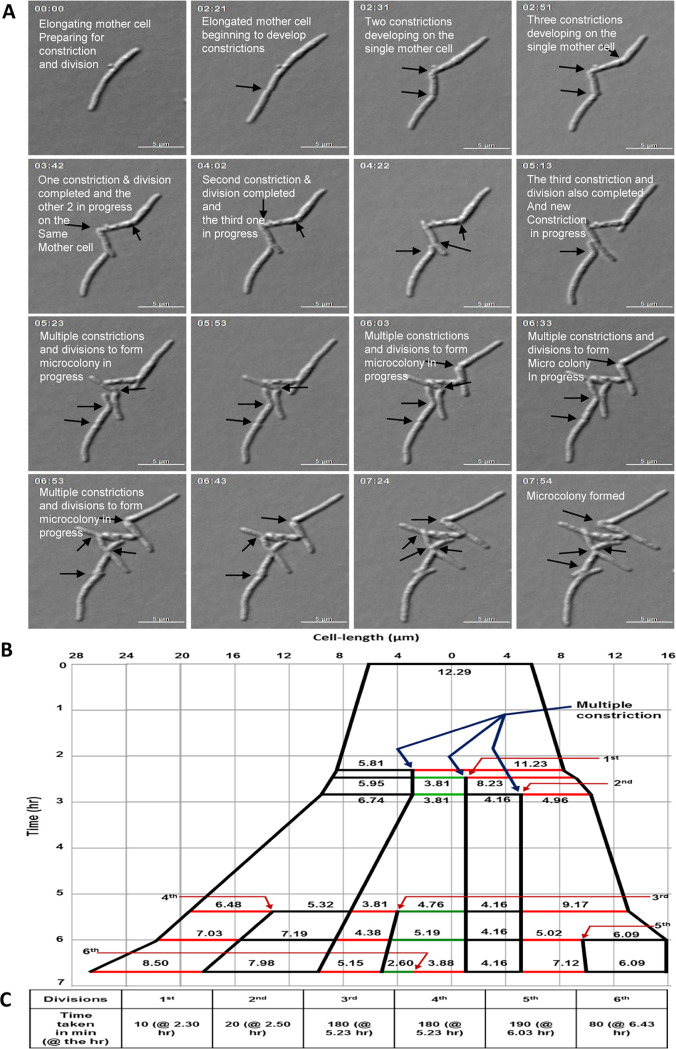
Live-cell time-lapse images and lineage of consecutive divisions of the 10× MBC rifampicin-exposed M. smegmatis cells from 90-h culture. (A) Live-cell time-lapse images. The arrows indicate the positions of multiple constrictions. These images were taken from Movie S1. (B) Lineage tracked from the live-cell time-lapse images of Movie S1. The zero time point does not correlate with the birth of the starting mother cell. Blue arrows indicate multiple constrictions. Red arrows indicate specific divisions. Different colored lines indicate different cells. The cell of length 4.16 μm (black line) did not grow or divide. The cell length was not drawn to scale. (C) Division duration of the individual cells between consecutive divisions, calculated from panel B by measuring the time taken from its birth to the next division.

Time-lapse imaging of the cells from the 90th h of 10× MBC rifampicin exposure (6 h before cell number spurt) confirmed multiple consecutive constrictions and divisions of the elongated multinucleated/septated cells to produce multiple sister-daughter cells (Fig. 5; derived from Movie S1). Tracking of the division lineages of these cells revealed consecutive divisions (Fig. 5B; derived from Fig. 5A). The duration between two consecutive divisions of the cells with multiple constrictions varied from 10 min to 190 min (Fig. 5C). Besides such variation in the duration between consecutive divisions of multiconstricted mother cells, one of the sister-daughter cells of some sister-daughter pairs did not show growth or division throughout the period we monitored. For instance, the 4.16-μm cell formed from the 2nd division, indicated by the black line in Fig. 5B, behaved in this manner. We observed elongated cells that developed a V-shaped bend indicating the impending “snapping model” of mycobacterial cell division (19, 30), characteristic of mycobacterial cells prior to constriction, but did not proceed to develop constrictions for division during the time monitored. The images from another live-cell time-lapse experiment using 10× MBC rifampicin-exposed M. smegmatis cells and tracking of their consecutive division lineages are provided in the miscellaneous files (see miscellaneous file Fig. MF1A and B; derived from the miscellaneous file Movie MF1 at https://doi.org/10.5061/dryad.9kd51c5fk). Here, also, the duration between consecutive divisions varied from 30 to 130 min (see miscellaneous file Fig. MF1C at https://doi.org/10.5061/dryad.9kd51c5fk). The live-cell time-lapse imaging of the M. smegmatis cells from the 96th h of the 2× MBC rifampicin-exposed cultures also showed multiple consecutive constrictions and divisions (Movie S2). The differential interference contrast (DIC) images derived from the Movie S2 are presented in the miscellaneous file (see Fig. MF2A at https://doi.org/10.5061/dryad.9kd51c5fk). Tracking the consecutive division lineage showed that the duration between two consecutive divisions of the cells with multiple constrictions varied from 20 min to 370 min (see miscellaneous file Fig. MF2B and C, derived from miscellaneous file Fig. MF2A at https://doi.org/10.5061/dryad.9kd51c5fk).

10.1128/mSphere.00994-20.8 MOVIE S1 90 h 10× MBC rifampicin-exposed M. smegmatis. Download Movie S1, AVI file, 15.1 MB. Copyright © 2020 Jakkala et al. 2020 Jakkala et al. This content is distributed under the terms of the Creative Commons Attribution 4.0 International license.

10.1128/mSphere.00994-20.9 MOVIE S2 90 h 2× MBC rifampicin-exposed M. smegmatis. Download Movie S2, AVI file, 4.4 MB. Copyright © 2020 Jakkala et al. 2020 Jakkala et al. This content is distributed under the terms of the Creative Commons Attribution 4.0 International license.

Thus, the rifampicin-surviving cells, formed from the M. smegmatis MLP cells exposed to 10× MBC rifampicin, showed an unusual cell division behavior generating a large number of sister-daughter cells within a short duration. The phenomenon seemed to have started off with the acquisition of *rpoB* RRDR mutations at high resister generation frequency, through the generation of elevated levels of hydroxyl radical, followed by multinucleated/septated elongation and multiple constriction, leading to multiple divisions to give high stochastic spurts in cell number. Having observed this phenomenon with 10× MBC rifampicin exposed cells, we wanted to verify whether the M. smegmatis cells would behave in the same manner when exposed to 1× MBC rifampicin.

### Stochastic high-cell-number spurts and resister generation frequency against 1× MBC rifampicin.

**FIG 6:**
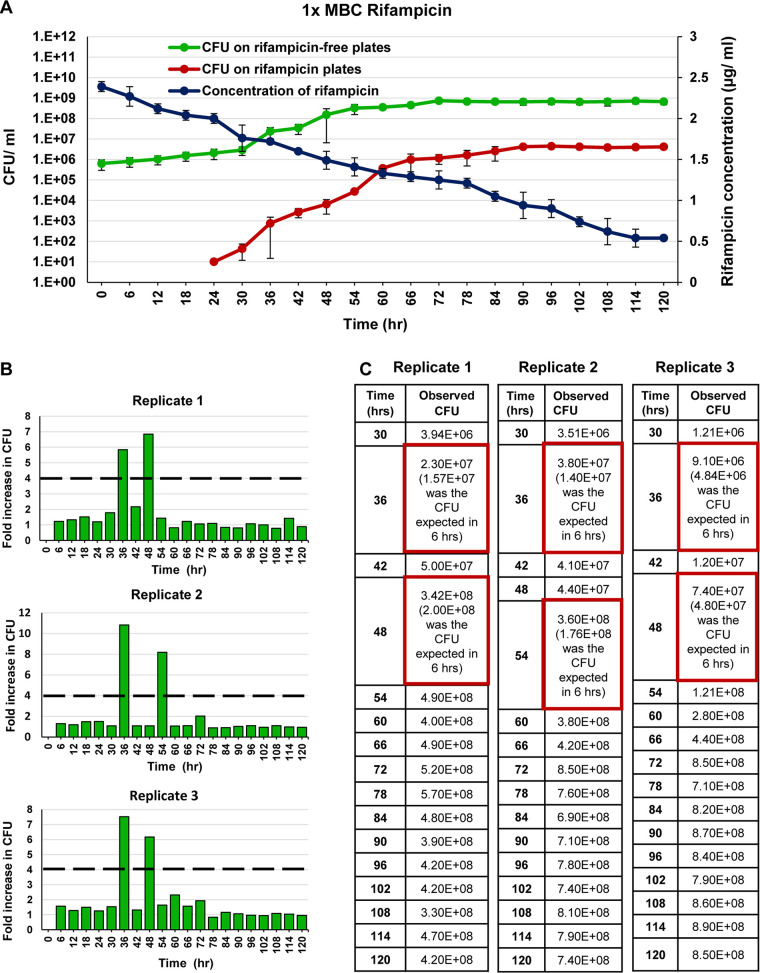
Response of M. smegmatis to 1× MBC rifampicin upon prolonged exposure. (A) The CFU from rifampicin-free plates, every 6 h during the exposure (green line), along with rifampicin levels in the medium (blue line). The resister generation is given by the red line. (B) The fold increase in the CFU, with respect to the CFU of the previous time point, was plotted for the biological triplicates. The dotted line indicates the expected 4-fold increase in the CFU in a 6-h period. (C) The observed CFU of the biological triplicates. The CFU spurts are given in red boxes with the expected CFU in parenthesis.

The response profile of M. smegmatis MLP cells (10^6^/ml) to 1× MBC rifampicin, as determined from the CFU on rifampicin-free plates, did not show a triphasic curve, and hence it was different from that against 10× MBC rifampicin. It showed a lag up to 30 h of exposure followed by a gradual rise up to 54 h before reaching a plateau, with a concomitant shallow and gradual decrease in the rifampicin concentration (Fig. 6A, green and blue lines, respectively). The CFU-readout based cell number spurts, which were 6- to 11-fold higher than the expected 4-fold, were found in one or the other 6-h periods among the biological triplicates (Fig. 6B and C). Unlike in the case of the exposure to 10× MBC rifampicin, the CFU spurts were low in number and occurred earlier during the exposure, with no CFU spurt after the 54 h of exposure (compare Fig. 6B and C with Fig. 1B and C).

In contrast, the CFU on the 50× MBC rifampicin plates showed a steady increase from 30 h onward until 60 h and then a shallow increase until 90 h, before reaching a plateau, indicating the emergence of rifampicin resisters (Fig. 6A, red line). From 60 h onward, the lower trajectory of the CFU profile from the rifampicin plates being almost parallel to that from the rifampicin-free plates indicated the emergence of a smaller number of resisters (Fig. 6A, green and red lines). This was unlike the response to 10× MBC rifampicin, where the CFU from the rifampicin plates and rifampicin-free plates overlapped from 60 h onward (Fig. 6A, green and red lines; compare with Fig. 1A, green and red lines). The CFU values showed 5- to 12-fold higher spurts from the 30- to 36-h period of exposure onward, with the number of CFU spurts of the resisters being more than those found on rifampicin-free plates (Fig. S4D and E; compare with Fig. 6B and C).

The rifampicin resister generation frequency of the cells from the 24-h and 30-h time points, which are the first time points of the appearance of resisters among the biological triplicates, were 4.5 × 10^−6^, 2.8 × 10^−6^, and 1.2 × 10^−5^ (Fig. S4F; see Fig. 6A, red line). These resister generation frequencies were ∼2 to 3 log_10_-fold higher than the natural resister generation frequency of 10^−8^ of M. smegmatis against rifampicin (11, 18) and about ∼2 to 3 log_10_-fold lower than that of the 10× MBC rifampicin cultures (compare Fig. S4F with Fig. 2C). This is consistent with the fact that antibiotic concentration influences the rate of mutation to antibiotic resistance and hence the resister generation frequency (31).

### Stochastic high-cell-number spurts and resister generation frequency against moxifloxacin.

A response like that shown against 10× MBC of rifampicin, was elicited by M. smegmatis cells against 10× and 3.75× MBC of moxifloxacin (1× MBC being 0.133 μg/ml for 10^8^ cells/ml; 10). The 10× MBC moxifloxacin-exposed culture showed a triphasic response with a killing phase, moxifloxacin surviving phase, and regrowth phase (Fig. S5A). The CFU of the M. smegmatis cells showed 5- to 14-fold high spurts during one or the other 6-h periods spanning the early regrowth phase (48 to 54 h) and beyond among the biological triplicates on the moxifloxacin-free plates (Fig. S5B and C). A similar response was shown by the triplicate cultures exposed to 3.75× MBC moxifloxacin, with 6- to 11-fold higher spurts in cell number during one or the other 6-h periods from the early regrowth phase (54 to 60 h) onward on moxifloxacin-free plates (Fig. S5D to F).

10.1128/mSphere.00994-20.5 FIG S5 (A to C) Response of M. smegmatis upon prolonged exposure to 10× MBC moxifloxacin. (A) CFU profile against 10× MBC moxifloxacin in culture, determined on moxifloxacin-free plates. The killing phase (0 to 36 h), the moxifloxacin-surviving phase (36 to 48 h), and the regrowth phase (48 to 120 h). (B) The fold change in the CFU once every 6 h. The dotted line indicates the expected 4-fold change in the CFU. (C) The CFU values at every 6-h interval. The CFU spurts in 6 h are shown in red boxes as the observed CFU and the expected 4-fold CFU in parenthesis. *n* = 3. (D to F) The response of the M. smegmatis upon prolonged exposure to 3.75× MBC moxifloxacin. (D) CFU profile from the moxifloxacin-exposed cultures determined on moxifloxacin-free plates. The killing phase (0 to 36 h), the moxifloxacin-surviving phase (36 to 54 h), and the regrowth phase (54 to 96 h). (E) The fold change in the CFU in 6-h periods. The dotted line indicates the expected 4-fold change in the CFU in 6-h periods. (F) The CFU values at every 6-h interval. The CFU spurts in 6 h are shown in red boxes, and the observed CFUs and the expected CFUs are in parenthesis *n* = 3. Download FIG S5, TIF file, 1.3 MB. Copyright © 2020 Jakkala et al. 2020 Jakkala et al. This content is distributed under the terms of the Creative Commons Attribution 4.0 International license.

In contrast, against 1× MBC moxifloxacin, the CFU showed a lag until 30 h of exposure, followed by a gradual rise until 78 h and a plateau (Fig. S6A). About 7- to 9-fold higher CFU spurts were found in one or the other 6-h periods among the biological triplicates on moxifloxacin-free plates (Fig. S6B and C). The number of CFU spurts against 1× MBC moxifloxacin was low and occurred much earlier, not after 54 h, unlike in the case of the 10× and 3.75× MBC moxifloxacin-exposed cells (Fig. S6B and C; compare with Fig. S5B, C, E, and F). Thus, the response profiles of M. smegmatis to 10× MBC, 3.75× MBC and 1× MBC moxifloxacin were similar to those against 10× MBC, 2× MBC, and 1× MBC rifampicin, respectively.

10.1128/mSphere.00994-20.6 FIG S6 (A and C) The response of M. smegmatis to 1× MBC moxifloxacin upon prolonged exposure. (A) The CFU profile from moxifloxacin-free plates, every 6 h during the exposure. (B) The fold increase in the CFU, with respect to the CFU of the previous time point of 6-h time intervals, was plotted for the biological triplicates. The dotted line indicates the expected 4-fold increase in the CFU in 6-h periods. (C) The observed CFU of the biological triplicates. The CFU spurts in 6 h are given in red boxes with the expected CFU values in parenthesis. *n* = 3. (D) Live cell imaging of the cells taken from the 54 h of 3.75× MBC moxifloxacin-treated culture. The cells show multiple septation. Arrows indicate constrictions. These images were taken from Movie S3. (E) Duration between consecutive divisions of multiple-constricted sister-daughter cells from Movie S3. The duration between the consecutive divisions of the multiple-constricted individual sister-daughter cell was calculated by measuring the time it took from the time of its birth to the next division. (F) Duration between consecutive divisions of multiple-constricted sister-daughter cells from Movies S1 and S3 and the miscellaneous file (see Movies MF1 to MF4 at https://doi.org/10.5061/dryad.9kd51c5fk). The duration between the consecutive divisions of the multiple-constricted individual sister-daughter cell was calculated by measuring the time it took from the time of its birth to the next division. Download FIG S6, TIF file, 2.1 MB. Copyright © 2020 Jakkala et al. 2020 Jakkala et al. This content is distributed under the terms of the Creative Commons Attribution 4.0 International license.

The live-cell time-lapse imaging and tracking of the lineage of multiple consecutive constrictions and divisions of 10× MBC moxifloxacin-exposed cells from the 54-h time point of exposure showed multiple constrictions and divisions (Movie S3 and the respective DIC images and division timings in Fig. S6D and E). Similarly, the live-cell time-lapse imaging of the M. smegmatis cells from the 54-h time point of 10× MBC moxifloxacin-exposed culture and from the 60 h time point of 10× MBC moxifloxacin-exposed replicate cultures are given in the miscellaneous files (see Movies MF 2 to 4 at https://doi.org/10.5061/dryad.9kd51c5fk). The corresponding tracking of the lineage of multiple consecutive constrictions and divisions of the movies MF 2 to 4 are given in the miscellaneous files (see Fig. MF3A to C, 4A to C, and 4D to F at https://doi.org/10.5061/dryad.9kd51c5fk). Calculation of the duration between consecutive divisions derived from Movies S1- to S3 (shown earlier) and the other movies (shown in the miscellaneous file Movies MF1 to 4 at https://doi.org/10.5061/dryad.9kd51c5fk) are represented in Fig. S6F. The duration between consecutive divisions showed variation from 10 min to 370 min (for Movies S1 to S3) and from 10 min to 250 min for the miscellaneous files (see Movies MF1 to MF4 at https://doi.org/10.5061/dryad.9kd51c5fk) (Fig. S6F). Thus, irrespective of the exposure to different MBC of different antibiotics and stochasticity in the timing of the cell number spurt, the duration between consecutive divisions of multiconstricted cells showed wide variation.

10.1128/mSphere.00994-20.10 MOVIE S3 54 h 10× MBC moxifloxacin-exposed M. smegmatis. Download Movie S3, AVI file, 9.7 MB. Copyright © 2020 Jakkala et al. 2020 Jakkala et al. This content is distributed under the terms of the Creative Commons Attribution 4.0 International license.

### Rifampicin/moxifloxacin-surviving M. tuberculosis populations showed high stochastic spurts in cell number.

**FIG 7:**
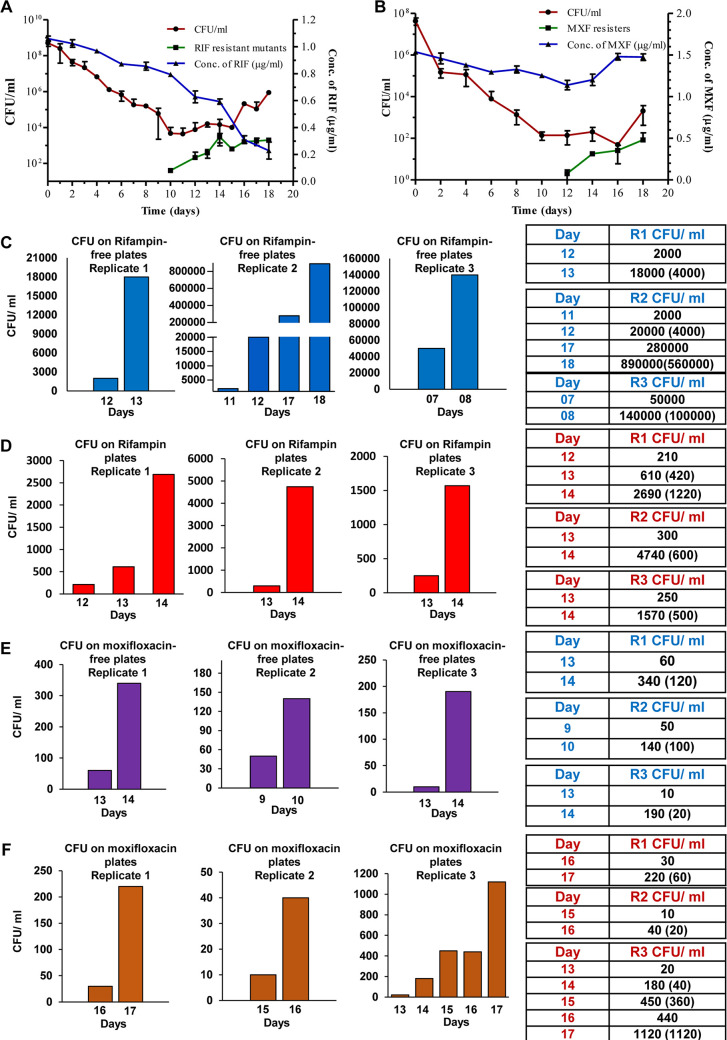
Susceptibility profiles and CFU spurts of M. tuberculosis upon prolonged exposure independently to rifampicin and moxifloxacin. (A and B). Susceptibility profile of M. tuberculosis cells to MBCs of (A) rifampicin and (B) moxifloxacin. (A) The susceptibility profile of M. tuberculosis cells, exposed to 10× MBC rifampicin (1 μg/ml) for 18 days, obtained by plating aliquots of the culture on rifampicin-free plate every day (●; red line) and in parallel on rifampicin-containing (50× MBC) plates (■; green line). The right-hand *y* axis shows the concentration of rifampicin during the exposure for 18 days (▲; blue line). (B) The susceptibility profile of M. tuberculosis cells, during exposure to 2× MBC moxifloxacin (1 μg/ml) for 18 days, obtained by plating aliquots of the culture on moxifloxacin-free plate every day (●; red line) and in parallel on moxifloxacin-containing (4× MBC) plates (■; green line). The right-hand *y* axis shows the moxifloxacin concentration during the entire exposure period (▲; blue line). Panels A and B are Fig. 1A and Fig. S2, respectively, from reference 9. (C to F) Observed CFU/ml (expected CFU/ml given in parenthesis considering the cell number doubling time to be 24 h) of M. tuberculosis cells during exposure to rifampicin (1 μg/ml; 10× MBC) or moxifloxacin (1 μg/ml; 2× MBC) at specific 24-h time points on antibiotic-free and antibiotic-containing (5 μg/ml rifampicin [50× MBC] or 2 μg/ml moxifloxacin [4× MBC]) Middlebrook 7H10 agar plates. CFU/ml on (C) rifampicin-free plates (blue bar) and (D) rifampicin plates (red bar graph), where the values are to be compared with the previous time point given in the graph. Similarly, CFU/ml on (E) moxifloxacin-free plates (purple) and (F) moxifloxacin plates (brown). The CFU values corresponded to the Fig. 1A (graph for rifampicin-exposed M. tuberculosis cells) and Fig. S2 (graph for moxifloxacin-exposed M. tuberculosis cells) in our earlier work (9), where the unusual spurt in the CFU went unnoticed and unreported by us. Only the time points showing CFU spurt, taken from our earlier published work (9), were reproduced here.

Earlier, we showed that the response of M. tuberculosis cells to bactericidal concentrations of rifampicin (10× MBC) and moxifloxacin (2× MBC), upon prolonged exposure for 20 days, involved killing, antibiotic-surviving (from day 10 to day 15 of rifampicin and from day 10 to day 16 of moxifloxacin), and regrowth phases (Fig. 7A and B, respectively). In light of the findings on M. smegmatis cells, examination of the CFU from the M. tuberculosis data revealed that the *de novo* emergence of rifampicin-resistant and moxifloxacin-resistant *rpoB* and *gyrA* mutants of M. tuberculosis regrowing from the respective antibiotic-surviving population also involved 3- to 20-fold high stochastic CFU spurts (Fig. 7C and D and E and F, respectively). The rifampicin resister generation frequency was 4 × 10^−2^, which was 8 log_10_-fold higher than that of the natural resister generation frequency of M. tuberculosis (10^−10^) against rifampicin *in vitro* (32). However, the CFU spurts went unnoticed by us and hence were not reported in our earlier paper (9). The high stochastic CFU spurts of the rifampicin/moxifloxacin*-*exposed M. tuberculosis cells also might have occurred through the formation of multinucleated/septated/constricted elongated cells regrowing from the respective antibiotic surviving population, followed by their multiple divisions producing a large number of sister-daughter cells. Here, also, earlier, we showed that the respective antibiotic-surviving populations generated elevated levels of hydroxyl radical inflicting genome wide mutations, from which the respective antibiotic genetic resisters got selected (9). The M. smegmatis also showed similar generation of hydroxyl radical and antibiotic resisters, which was reported by us earlier (10, 11). Thus, both M. smegmatis and M. tuberculosis showed comparable cell division behavior in their response to different MBC of antibiotics upon prolonged exposure.

### Gene expression profile of the M. smegmatis regrowing population hinted heterogeneity.

We determined the profile of the genes expressing in the M. smegmatis population regrowing from the antibiotic-surviving phase using quantitative reverse transcriptase PCR (qRT-PCR). The total RNA of high structural integrity and purity prepared from the biological replicate samples at 6-h intervals from the 66th h to 114th h of rifampicin exposure was used. The genes were selected from cell division, DNA replication and repair, *whiB* family transcription factors, and SOS regulons. Various extents of expression were found for all the selected genes between the biological replicates (sets 1 and 2), indicating that the regrowing population contained cells with high levels of heterogeneity in terms of their gene expression profile (Fig. S7A). It is possible that the proportions of the regrowing/nongrowing cells of different phenotypes and the specific stages of regrowth might vary among the replicates. Thus, overall, it was difficult to make any meaningful conclusion from the qRT-PCR data. The profiles gave an indication of high levels of heterogeneity in terms of stages of the cells in their regrowth and antibiotic tolerance.

10.1128/mSphere.00994-20.7 FIG S7 (A) Gene expression profile determined using qRT-PCR. The expression profile of the selected genes from the set 1 and set 2 of each gene at specific time points from 66 h to 114 h of exposure. *n* = 2. The profile of the total RNA used for qRT-PCR is shown below. (B) Oligonucleotide primers used in the study. Download FIG S7, TIF file, 1.8 MB. Copyright © 2020 Jakkala et al. 2020 Jakkala et al. This content is distributed under the terms of the Creative Commons Attribution 4.0 International license.

### The unique cell division behavior of the antibiotic resister clones emerging *de novo* from the antibiotic-surviving population.

**FIG 8:**
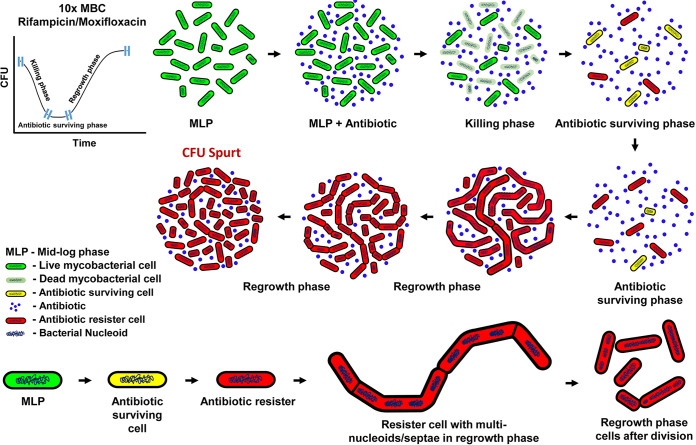
The model for the unique cell division behavior of *de novo*-emerged rifampicin/moxifloxacin resisters. The model depicting the physiological mechanisms, involving acquisition of antibiotic target mutation, multinucleation, and multiseptation leading to multiple constrictions to give CFU spurts, which drive the emergence of antibiotic resisters from the antibiotic-surviving populations in the M. smegmatis and M. tuberculosis cultures exposed to high-MBC rifampicin or moxifloxacin for prolonged duration.

Thus, the observations of the M. smegmatis and M. tuberculosis cells exposed to high/low MBC of rifampicin and moxifloxacin for prolonged duration revealed that the respective antibiotic-surviving populations generated elevated levels of hydroxyl radical, which inflicted genome-wide mutations. The cells that acquired mutation against the antibiotic target got selected and began to grow and divide through multinucleation/septation/constriction. The stochastic multiple divisions at the multiple constrictions at various times generated an unusually large number of sister-daughter cells in the liquid cultures in the continued presence of high MBC of the respective antibiotic. The sequence of these physiological events, driving the emergence of a large number of antibiotic resisters from the antibiotic-surviving population is graphically presented in the Fig. 8.

## DISCUSSION

### Multinucleated/septated/constricted division of antibiotic resisters.

The present study delineated the dynamic physiological mechanisms behind the multiplication of genetic resister clones, which emerged *de novo* from the M. smegmatis*/*M. tuberculosis cells surviving in the presence of bactericidal concentrations of rifampicin/moxifloxacin *in vitro*. Bacteria exposed to stress agents, including antibiotics, adopt the common strategy of cell-division arrest, acquisition of mutations, and regrowth, forming multinucleated/septated elongated/filamented cells that divide at multiple constrictions, forming an unusually large number of mono-nucleated cells causing cell number spurts (reviewed in reference 33). For instance, E. coli W3110/MG1655 stationary-phase persister cells, when exposed to sub-MIC ciprofloxacin or ofloxacin (references 27 and 34, respectively), became multinucleated elongated/filamented, and upon withdrawal of the antibiotic, divided synchronously, generating a large number of sister-daughter cells. However, in the continued presence of ciprofloxacin, progeny resister cells budded from the filamentous cells, with 250-fold higher resister generation frequency (27). The authors proposed that the formation of multiple nucleoids enabled the generation of mutant chromosomes through SOS-driven mechanisms and possible recombination of new alleles between chromosomes to generate antibiotic-resistant bacteria (27).

### Merits of multinucleated/septated/constricted division of antibiotic resisters.

Exposure to antibiotics generates the DNA-nonspecific mutagen, hydroxyl radical, which inflicts genome-wide random mutations (20–22). Therefore, the cost of acquiring an antibiotic-resistant mutation often involves moderate to severe compromise on growth characteristics (35). Therefore, it is necessary for the bacilli to acquire mutation and escape from the antibiotic exposure-generated oxidative and the antibiotic-target-specific stress and establish a resister population. In this regard, the multinucleation/septation followed by multiple constrictions and divisions generating a large number of antibiotic-resistant sister-daughter cells from each mother cell seemed be a reliable strategy to establish an antibiotic-resister population.

If the multinucleation had occurred after the mutation acquisition, the same mutations would be present in all the nucleoids. However, if the mutagenesis had occurred after multinucleation, possible recombination among new allelic chromosomes would spread the mutation to other nucleoids. However, in bacterial systems with low recombination frequency, the possibility of mutations spreading across the multinucleoids might also be low. In such a case, each sister-daughter nucleoid would have a unique genomic mutational profile. This could increase the chances of survivability of a higher number of the individual sister-daughter cells inheriting the nucleoid with the unique genomic mutational profile. These possibilities give the multinucleated cells an advantage in resister generation.

### Precursors of the genetic resister clones in the antibiotic survivors.

The emergence of large number of M. smegmatis*/*M. tuberculosis antibiotic genetic resisters was preceded by the presence of subpopulations surviving in the continued presence of lethal concentrations of rifampicin/moxifloxacin, as demonstrated by us in the present study and earlier (9–11). It implied that these subpopulations contained rifampicin-tolerant cells, as defined (16), classical persisters, as termed (4, 5), and several other phenotypic subpopulations that might survive using several other mechanisms (2, 7). We did not “remove and reintroduce” rifampicin/moxifloxacin on the antibiotic-surviving subpopulations to verify whether the cells in this phase contained classical persisters, as termed in the original work on the identification of persisters (4) and as categorized recently (5). Further, we did not either test whether the cells in these subpopulations behaved as per the definition of “antibiotic-tolerant” cells (16). Methodologies to segregate these different types of antibiotic-tolerant subpopulations are necessary to trace out the original precursors of the cells that acquired mutations and adopted multinucleation/septation/constriction that facilitated abrupt division generating a large number of antibiotic-resistant sister-daughter cells.

### The probable sequence of events that led to cell number spurts.

The M. smegmatis antibiotic resisters started emerging from the respective antibiotic-surviving phase at 36 h of the rifampicin-exposed cultures and 30 h of the moxifloxacin-exposed cultures in the present and earlier studies (10, 11). The regrowth of the cells started from 48 h and 54 h, respectively, for the rifampicin- and moxifloxacin-exposed M. smegmatis cultures (10, 11). Similarly, the genetic resisters of M. tuberculosis against rifampicin and moxifloxacin emerged *de novo* from the respective antibiotic surviving phase of day 10 and day 12, of the respective cultures (9; see Fig. 7A and B). The regrowth of these M. tuberculosis cultures occurred from day 15 and 16, respectively (9; see Fig. 7A and B). These observations implied that the genetic resister clones to the respective antibiotic emerged from the respective antibiotic-surviving populations. The multinucleation/septation occurred in the cells in the respective regrowing phase that ensued the antibiotic surviving phase. Thus, the regrowth occurred most likely in the cells that have already acquired antibiotic target-specific mutation. The elevated levels of hydroxyl radical in the antibiotic-surviving population and their decline in the regrowth phase population supported this possibility. The reciprocal relationship between the hydroxyl radical levels and regrowth CFU (see Fig. S1B) in the continued presence of rifampicin also supported this possibility. Thus, the multinucleation/septation/constriction followed by cell number spurts might have occurred in the cells that were already carrying mutations.

### The features of the cell number spurt phenomenon.

The spurts in cell number indicated by the CFU spurts at different 6-h periods in each replicate culture implied that the CFU might have been formed from different cells and not through the repeated multiplication of the same cells carried over from one period to the next during the continuous culture. The low and various CFU values just prior to the CFU spurt at different time points supported this possibility. In contrast to the CFU spurts, the CFU for many consecutive 6-h durations in the regrowth phase have not shown even the expected 4-fold rise (see Fig. 1B and C; *n* = 3). It indicated that either all the cells in the regrowth phase have not undergone division or that the cells might have undergone division with shorter/longer duration between consecutive divisions in the multiconstricted cells. In fact, live-cell time-lapse imaging of the growth and division of regrowth phase cells showed that many cells grew and divided at multiple constrictions at various intervals anywhere from 10 min to 370 min between consecutive divisions of the multiconstricted cells. This variation in the division time intervals was caused by the consecutive divisions, at multiple constrictions in each of the multiconstricted cells, occurring at different times, in an asynchronous manner. Further, during the period monitored, some cells grew at a slow rate and divided at multiple constrictions at longer intervals, while some cells did not grow at all.

### Formation of resisters against high- and low-MBC rifampicin/moxifloxacin.

Antibiotic resisters could be selected upon prolonged exposure to high and low MBC of rifampicin (10×, 2×, and 1×) and moxifloxacin (10×, 3.75×, and 1×). Against 1× MBC rifampicin/moxifloxacin, the acquisition of genetic mutations might have occurred during the phase with a shallow increase in the CFU, enabling the mutants to grow and divide, causing a steady rise in the cell number. The first difference between the responses to 10× and 1× MBC rifampicin was that the resisters began to emerge at 24 h of exposure, unlike at 36 h of exposure in the 10× MBC rifampicin-exposed cultures (compare the red lines in Fig. 6A and Fig. 1A). Second, the resister generation frequency against 1× MBC rifampicin was lower by 2-log_10_-fold (∼10^−5^, compared to ∼10^−3^ in the case of exposure to 10× MBC rifampicin). It is known that antibiotic resisters, with high resistance mutations, can emerge *de novo* against low concentrations of antibiotics, get rapidly enriched, and contribute to stepwise development of antibiotic resistance and are generally more fit than the resisters selected against high concentrations (3, 36). Thus, the emergence of resisters against 10× and 1× MBC rifampicin in liquid cultures and the presence of high resisters on 50× MBC rifampicin plates from both the M. smegmatis cultures were consistent with these observations.

### M. tuberculosis CFU rise in the antibiotic-exposed tuberculosis patients/animal models.

A rise in the tubercle bacilli CFU against antibiotics has been noted in tuberculosis patients and in infected mice and macrophages. The tuberculosis patients, who had infection with streptomycin-sensitive M. tuberculosis strains and were under treatment for 6 or more months, were found to harbor a 4 to 4,000 times higher number of streptomycin-resistant strains than that of the control strain, M. tuberculosis H_37_R_v_ from the 42nd day of treatment onward (37). Similarly, the relapse of tubercle bacilli in the 14-week rifampin/isoniazid/pyrazinamide-exposed modified Cornell mouse model was suggested to be from the antibiotic-surviving bacilli (38). The observation of chromosome equivalents (CEQs) being always 2- to 3-fold higher than the respective CFU of M. tuberculosis in chronically infected mouse lungs might have been due to the presence of multinucleated elongated bacilli (39). Similarly, the CFU per lung of M. tuberculosis H_37_R_v_-infected nude mice treated with rifampicin/isoniazid (INH) dropped from ∼10^8^ to ∼10^5^ in 2 months, followed by an increase to ∼10^7^ and ∼10^9^ CFU per lung on the 3rd month and 4th month, respectively, yielding INH-resistant strains (40). The CFU of M. tuberculosis H_37_R_v_ from infected macrophages, exposed to rifapentine/rifampin also showed a rapid decrease during the first 7 days, followed by a plateau for about 7 days and a subsequent gradual increase despite a high concentration of the antibiotic (41). In all these studies, it was quite possible that the CFU rise might have occurred from multiple divisions of multinucleated, multiseptated antibiotic-resistant bacilli. If this is the case, then the response of the bacilli with a sudden spurt in cell number is an inherent trait of the antibiotic-stressed mycobacteria irrespective of their living habitat and pathogenic/nonpathogenic status.

## MATERIALS AND METHODS

### Bacterial strains and culture conditions.

Mycobacterium smegmatis mc^2^155 (42) was grown in autoclaved sterilized fresh Middlebrook 7H9 liquid cultures containing 0.2% glycerol (Fisher Scientific) and 0.05% Tween 80 (Sigma), in the presence of rifampicin or moxifloxacin, under continuous shaking at 170 rpm and 37°C by taking a 5:1 headspace to volume ratio. Plating experiments were performed on *Mycobacterium* 7H11 agar plates (Difco), with rifampicin (125 μg/ml; MP Biomedicals) or without rifampicin, at 37°C for 3 to 4 days. All the experiments were conducted using actively growing mid-log-phase cells with an approximate cell density of 10^6^ cells/ml.

### Antibiotic response profile determination.

From the start of the experiment, liquid cultures were exposed to 25 μg/ml (10× MBC)—2.5 μg/ml (1× MBC) concentration of the antibiotic, rifampicin (MP Biomedicals). The antibiotic stock solution (50 mg/ml) was made by dissolving rifampicin powder (MP Biomedicals) in dimethyl sulfoxide (DMSO; Merck Millipore) and was filter sterilized using 0.22-μm polyvinylidene difluoride (PVDF) syringe filters (Millex-GV). Dilutions of the antibiotic (rifampicin) were performed using 0.22-μm PVDF (Millex-GV) filter-sterilized DMSO (Merck Millipore). Similarly, moxifloxacin solutions (Cayman Chemicals; 0.5 μg/ml, i.e., 3.75× MBC; 1.5 μg/ml, i.e., 10× MBC; 0.15 μg/ml, i.e., 1× MBC; 10) were prepared from the stock of 2 mg/ml solution in DMSO. The appropriate dilution of moxifloxacin was added to an independent 100-ml M. smegmatis secondary culture. Post-antibiotic exposure, the cells were taken at every 6-h time interval and plated on antibiotic-free and antibiotic-containing (125 μg/ml) *Mycobacterium* 7H11 agar (Difco) plates. The total number of cells present at the specific time points and their respective resisters in the culture were determined from these CFU on these plates. Dilution of the cultures for the plating was performed using fresh Middlebrook 7H9 medium. Plates were incubated (Innova 4200 incubator shaker) at 37°C for 3 to 5 days.

### Calculation of fold increase in the CFU.

The fold increase in CFU at any specific time point was calculated by normalizing the CFU value of that specific time point with the CFU obtained at its previous time point.

### Rifampicin bioactivity assay.

Rifampicin bioactivity was monitored, as described (43), but with minor modifications, as we had performed for the M. tuberculosis cultures (9). In brief, rifampicin-sensitive Staphylococcus aureus (ATCC 25923) was embedded LB agar medium and poured into petri plates. Once the medium was solidified, wells were made of 0.5 cm diameter. Then, 50 μl of the culture supernatant was added to the wells and incubated at 37°C overnight. A vernier caliper was used to measure the zone of growth inhibition. For calculating the unknown concentrations of the rifampicin at different time points, a standard graph was made with known concentrations of rifampicin to get the zone of growth inhibition.

### HPF staining of M. smegmatis cells.

HPF staining of cells post-antibiotic exposure (10× MBC rifampicin; Sigma) was carried out by taking 500 μl of the culture from respective time points into Eppendorf tubes and incubating it with 5 μM 3′-(p-hydroxyphenyl fluorescein) (HPF; Invitrogen) (0.5 μl of HPF from 5 mM stock) (44, 45) at 37°C for 15 min with shaking at 170 rpm in a bacteriological incubator shaker (Innova 4200 incubator shaker) in the dark. The cells, postincubation with HPF, were centrifuged at ∼5,000 × *g* for 10 min at room temperature and resuspended in 200 μl of fresh Middlebrook 7H9 (Difco), and the cell suspension was transferred into a clear-bottom black multiwell polystyrene assay plate. Fluorescence was observed using a Tecan Infinite 200 PRO series plate reader at the excitation maxima of 490 nm and emission maxima of 520 nm. The fluorescence obtained by the multiplate reader was divided with by CFU value obtained by plating to get the fluorescence value per cell. The obtained value was normalized with the value from the 48-h time point, and the fold increase in the fluorescence per cell was plotted in the graph against time.

### Hoechst staining of nucleoids.

The staining of nucleoids was performed as per the instructions from the manufacturer (Molecular Probes/Invitrogen). First, 200 μl of the cells from the mid-log as well as from the regrowth phase were put into an Eppendorf tube and pelleted down at ∼5,000 × *g* for 10 min at room temperature. The supernatant was removed, and the cell pellet was washed once with fresh Middlebrook 7H9 (Difco) medium and pelleted again with the same centrifugation conditions. The cell pellet was resuspended in 200 μl of fresh Middlebrook 7H9 (Difco) medium, and 10 μg/ml concentration of the Hoechst 33342 stain (Sigma) was added. This mixture was incubated at 37°C (Innova 4200 incubator shaker) for 10 min in the dark. After 10 min, the cells were washed to remove the excess dye by pelleting down at ∼5,000 × *g* for 10 min at room temperature, the pellet was resuspended in fresh Middlebrook 7H9 (Difco) medium, and a drop of the cell suspension was placed on a clean glass slide and dispersed evenly. The slide was air dried at room temperature for 10 min in the dark, and a small drop of glycerol was added and covered with coverslip. The slide thus made was observed under a Carl Zeiss AXIO Imager M1 microscope at ×100 magnification by putting a drop of immersion oil on top of the coverslip at a wavelength of excitation maximum of 350 nm and emission maximum of 461 nm. Every bi-lobular blue fluorescence image was considered one nucleoid, and each spread-out blue fluorescence image per cell was also considered one nucleoid.

### Atomic force microscopy.

Sample preparation for atomic force microscopy was carried out as described (46) with minor modifications. In brief, 1 ml of the cells from the regrowth phase (96 h) were placed in a fresh Eppendorf tube and harvested by centrifugation at ∼5,000 × *g* for 10 min at room temperature. The cell pellet was washed once with fresh Middlebrook 7H9 liquid medium. A glass coverslip was cleaned once with acetone (Merck Millipore), and after drying, a drop of the cell suspension was placed on the coverslip. The coverslip was air dried for 10 min at room temperature, and post-air drying, it was washed gently with fresh deionized water and again set for drying for 10 min at room temperature. The cells fixed on the glass coverslip were observed under an atomic force microscope in a contact-independent mode of scanning.

### Transmission electron microscopy.

Cells from the regrowth phase (96 h) were taken and processed for transmission electron microscopic analysis as described (47), with minor modifications (48). In brief, 1 ml of the cells from the regrowth phase (96 h) was placed in an Eppendorf tube and harvested by centrifugation at ∼5,000 × *g* for 10 min at room temperature. Supernatant was discarded, and the cell pellet was resuspended in 0.15 M sodium cacodylate-HCl buffer, pH 7.4, containing 1% osmium tetroxide (OsO_4_) (wt/vol) (Sigma) and incubated (Innova 4200 incubator shaker) for 1 h at room temperature in the dark. After 1 h, cells were washed with the same buffer by pelleting them at ∼5,000 × *g* for 10 min at 25°C. The cell pellet was resuspended in the same buffer containing 2% glutaraldehyde (vol/vol) (Sigma) and 2% tannic acid (wt/vol) and incubated at 25°C for 2 h in the dark. The cell suspension was washed with the same buffer and centrifuged for 10 min at room temperature at ∼5,000 × *g*, and the pellet was resuspended in the same buffer containing 1% OsO4 and incubated overnight at 4°C. Post-overnight incubation, the cell suspension was washed with the same buffer at ∼5,000 × *g* for 10 min at 25°C, and the pellet was subjected to dehydration with serial dilutions of ethanol (Merck Millipore) (20%, 30%, 50%, 70%, 90%, 100%, and 100%). After dehydration, the cell pellet was resuspended in 50% LR White resin (1:1 volume of LR White resin [London Resin Company] and 95% ethanol [Merck Millipore]), and the cell suspension was stored at 4°C. Gelatin blocks were prepared by spinning down this cell suspension at 25°C for 10 min at ∼5,000 × *g*, and an aliquot of the cell pellet was taken and adhered it to the bottom of the gelatin capsule (Electron Microscopy Sciences, USA), which was filled with 100% LR White resin. This gelatin capsule was kept at 60°C for 48 to 56 h until it became rock hard. The blocks were trimmed and cut into 70-nm ultrathin sections using an ultramicrotome (Power Tome XI). The sections were collected on a copper grid (150 mesh by 165 μm pitch; Sigma-Aldrich), and the copper grids containing sections were subjected to uranyl acetate and led citrate staining. The stained sections were observed at 120 kV under transmission electron microscopy (Tecnai Bio-TWIN).

### Glycerol estimation.

Free glycerol concentration in the culture supernatant was measured by converting glycerol to formaldehyde using periodate (49) and spectrophotometric estimation of formaldehyde at 410 nm after Hantzsch reaction (50, 51). The glycerol concentration in the supernatant was measured at different time points as described (51), with minor modifications. In brief, the reagents were prepared as follows: (i) acetic acid stock solution: 1.6 M aqueous solution in autoclaved distilled water; (ii) ammonium acetate stock solution: 4.0 M aqueous solution in autoclaved distilled water; (iii) 0.2 M acetyl acetone solution: 200 μl of acetyl acetone was added into 5 ml of acetic acid stock solution and mixed well, and 5 ml of ammonium acetate stock solution was added; (iv) 10 mM sodium (meta) periodate solution: 21 mg of sodium (meta) periodate was dissolved in 5 ml of acetic acid stock solution, and once the sodium (meta) periodate was completely dissolved, 5 ml of ammonium acetate stock solution was added. The working solvent comprised equal volumes of autoclaved distilled water and 95% ethanol.

Different concentrations of glycerol standard were prepared as 0.4, 0.2, 0.1, 0.05, 0.025, 0.0125, and 0.00625% by serial dilution using distilled water. Then 13 μl of glycerol standard was dissolved in 117 μl of distilled water. Next, 390 μl of working solvent was added to 130 μl of the diluted standard, and 312 μl of sodium (meta) periodate solution was added and mixed on a vortex mixer for 30 sec. Subsequently, 312 μl of 0.2 M acetyl acetone solution was added and kept in a heating block at 70°C for 1 min. The sample was then immediately cooled by keeping the tube in ice. Absorbance was measured immediately at 410 nm in a UV-visible spectrophotometer. The standard calibration curve was constructed, and the equation for the straight line, i.e., *y* = *mx* + *c*, was used to determine the glycerol concentration in the culture medium, where *y* is the absorbance at 410 nm, *m* is the slope, *c* is the *y* intercept, and *x* is the unknown concentration of glycerol to be determined as *x* = (*y* – *c*) ÷ *m*.

The glycerol concentration in the culture supernatants of the antibiotic-exposed culture was determined. In brief, culture supernatants at different time points were collected by centrifuging 1 ml of M. smegmatis culture at ∼5,000 × *g* for 10 min at room temperature, from independently grown biological triplicate cultures. Samples were treated with sodium (meta) periodate and acetyl acetone solution in the same way as in the case of the standard. Absorbance was measured at 410 nm, and the glycerol concentration (*x*) was calculated with the equation of the straight line of the standard calibration curve.

### Expected and observed CFU.

The fold change of CFU at any specific time point was calculated by considering the next time point CFU value as “B” and subtracted this value from the earlier time point CFU value (considered “A”), and the resulting value was divided by the time point A. The expected CFU increase within 6 h will be 4 times the number of cells present at the previous time point. Thus, any time point where the cells showed more than a 4-fold increase in CFU was considered an unusual division. The table was made accordingly with the observed and expected CFU values. The time points where the cell number was higher than the expected CFU were highlighted in the box.

### Live-cell time-lapse microscopy.

Live-cell time-lapse microscopy of M. smegmatis regrowth-phase cells was performed using the agarose pad method as described (52, 53), with minor modifications. An agarose pad was prepared by using spent medium of 90-h rifampin (25 μg/ml), 60 h and 54 h of moxifloxacin (1.5 μg/ml)-exposed culture, and 1.75% low-melting-point agarose on a clean glass slide. After solidification, a portion of the agarose (about one-fifth of the total agarose pad area) was cut out using a blade to make a well for the introduction of rifampicin into the agarose pad. Toward one side of the well, a tip was attached which was connected to a syringe for removal of rifampicin-containing medium. Then, 10 μl of 90-h rifampin (25 μg/ml) and 60 and 54 h of moxifloxacin (1.5 μg/ml)-exposed M. smegmatis cells were placed on top of the agarose pad and spread evenly by tilting the slide. The slide was covered with a coverslip at a 45° angle from the base of the glass slide, leaving a portion of the well open for the introduction of rifampicin with a syringe. The agarose pad with the cells was kept at 37°C for 1 h incubation to facilitate cell adhesion. This slide was observed under a Carl Zeiss AxioVision 4 microscope using the live-cell imaging option, at ×100 magnification (DIC), with 0.2-μm slice distance Z-stacking at 37°C. Individual cells were observed, and the DIC images were taken every 10 min. The data were analyzed, and the parameters such as cell length and cell constriction were determined on the images, using AxioVision 4 and ImageJ software.

### Genomic DNA isolation.

Genomic DNA was prepared from the MLP and from the rifampicin-exposed cell cultures using the phenol-chloroform extraction method. In brief, the cell pellet was lysed by resuspending in 1 ml of Tris-HCl-EDA buffer (10 mM Tris-HCl containing 1 mM EDTA, pH 8) containing 3 mg/ml lysozyme (chicken egg white; Fluka) and 1 mg/ml lipase (from Candida cylindracea; Sigma) in an Eppendorf tube. This cell suspension was kept in a 37°C water bath for 4 h with intermittent mixing by inverting the Eppendorf tubes. The cell lysis was carried out by adding 2% SDS (Sigma) (final concentration) and incubated at 55°C for 10 min again with intermittent mixing (once every 30 min) by inverting the tubes. The cell lysate was centrifuged at ∼7,800 × *g* for 10 min at 4°C. The supernatant (∼800 μl) was collected using cut tips and subjected to phenol-chloroform extraction using equal volumes of an ice-chilled phenol (Tris-HCl saturated, pH 7.8; Sisco Research Laboratories) chloroform (Nice Chemicals) mixture in a 1:1 ratio (volume to volume). The suspension was mixed by inverting the Eppendorf tube ∼5 to 10 times and centrifuged at ∼7,800 × *g* for 10 min at 4°C. The supernatant was collected and mixed with an equal volume of phenol (Tris-HCl saturated; pH 7.8) and again mixed by inverting the Eppendorf tubes ∼5 to 10 times. This suspension was centrifuged at ∼7,800 × *g* for 10 min at 4°C. To the supernatant, an equal volume of ice-chilled chloroform was added and mixed again by inverting the tubes 5 to 10 times. This mixture was again centrifuged at ∼5,000 × *g* for 10 min at 4°C, and the supernatant was collected. To this supernatant, a one-tenth volume of 0.3 M sodium acetate (pH 7.4; Sigma) (final concentration) was added and mixed well with a pipette. After this, 2.5 volumes of 95% ice-cold ethanol (Merck Millipore) were added and mixed vigorously by inverting the tubes 15 to 20 times. The suspension was kept at 4°C overnight for better precipitation. After overnight incubation at 4°C, DNA was pelleted down by centrifuging the suspension at ∼11,300 × *g* for 20 min at 4°C. Supernatant was discarded, the cell pellet was washed once with 70% ethanol (Merck Millipore), and the pellet was air dried for 10 min. After 10 min, the DNA pellet was dissolved in 1× Tris-HCl-EDTA buffer (10 mM Tris-HCl, 1 mM EDTA, pH 8). This DNA was subjected to RNase A treatment by incubation with 1 μl of 10 mg/ml RNase A (bovine pancreatic RNase; Sigma) at 50°C for 60 min. After RNase treatment, DNA was reextracted with phenol-chloroform and precipitated as mentioned earlier. Finally, the genomic DNA (gDNA) obtained was dissolved in 1× Tris-HCl-EDTA buffer (10 mM Tris-HCl, 1 mM EDTA, pH 8) and stored at 4°C.

### PCR amplification of RRDR sequence and DNA sequencing.

The RRDR locus was PCR amplified using RRDR-specific forward and reverse primers (Fig. S7B) using Phusion DNA polymerase (Thermo Fisher Scientific, USA). The PCR-amplified products were run on 1.5% agarose gel, and the specific amplified band was eluted using a gel elution kit (GeneJET gel extraction kit; Thermo Fisher Scientific, USA). Sequence determination was performed for both the strands of the DNA by Chromous Biotech, Bangalore, India.

### Total RNA extraction from the rifampicin-exposed regrowth-phase cells.

Total RNA extraction was performed using the hot-phenol method (54) with slight modifications. Cell pellets obtained from different time points were centrifuged at 5,000 × *g* for 10 min at 4°C, snap-frozen using liquid nitrogen, and stored at –70°C. The pellet was crushed using a micropestle in liquid nitrogen and mixed with 1 ml of lysis buffer (sodium acetate 100 mM, pH 5.2; EDTA 10 mM, pH 8.0; vanadyl ribonucleoside complex [VRC], 5 mM; and 1% sodium dodecyl sulfate [SDS] in RNase-free double-distilled water). Equal volumes of cell lysate and hot phenol (phenol saturated with 0.1 M sodium acetate, pH 5.2, and prewarmed at 65°C) were mixed and vortexed intermittently by keeping them at 65°C for 10 min. After such extraction for 10 min, the samples were kept on ice and then centrifuged at 12,000 × *g* for 10 min at 4°C. The aqueous layer was extracted twice more with an equal volume of hot phenol. Postcentrifugation, the aqueous phase was collected, mixed with an equal volume of a 1:1 (vol/vol) ratio of ice-cold phenol:chloroform, vortexed well, and centrifuged. This was repeated twice more. Finally, the aqueous phase was collected, and an equal volume of ice-cold chloroform was added, vortexed well, and centrifuged at 12,000 × *g* for 10 min at 4°C. This procedure was repeated twice more. To the aqueous phase, a final concentration of 0.3 M sodium acetate, pH 5.2, and 2.5 volume of 95% ice-cold ethanol was added and kept for precipitation overnight at –70°C. Following overnight precipitation, the samples were centrifuged at 12,000 × *g* for 10 min at 4°C. The pellet thus obtained was washed with 70% ethanol twice and kept for air drying. The air-dried pellet was dissolved in RNase-free water, and the total RNA was quantitated using a nano-spectrophotometer.

The RNA sample was treated with DNase I (50 U/μl; Thermo Fisher Scientific) to remove any DNA contamination, using manufacturer’s protocol. DNase treatment was performed by taking 10 μg of RNA sample and treating it with 10 U of DNase I in the presence of 10× reaction buffer with MgCl_2_ (provided by the manufacturer) at 37°C for 45 min. The DNase I was removed from the sample using phenol chloroform extraction. The aqueous phase collected was mixed with 0.3 M sodium acetate (pH 5.2) and 2.5 volume of 95% ice-cold ethanol and kept for overnight precipitation at –70°C. Subsequently, the sample was centrifuged at 12,000 × *g* for 10 min at 4°C. The pellet was washed with 70% ethanol, centrifuged again at the same condition, air-dried, dissolved in RNase-free water, and quantitated using a NanoDrop 2000 spectrophotometer.

The RNA integrity was checked by taking 1 μg of total RNA in the RNA loading buffer containing 10 μl of formamide (Sigma), 3.2 μl of formaldehyde solution (Merk), 2 μl of RNA loading dye, and 0.5 μl of 5 μg/μl ethidium bromide. The RNA loading dye used in the study contains 200 μl of glycerol (Merk), 20 mM sodium borate (pH 8.3), 2 mM EDTA (pH 8.0), bromophenol blue, and xylene cyanol in RNase-free water. PCR was performed using M. smegmatis 16S rRNA primers (Fig. S7B) to confirm the absence of DNA in the RNA sample. The total RNA integrity was checked on 1% denaturing formaldehyde agarose gel (7.5 by 5.0 cm) containing 1% agarose (Sigma), 0.5 mM EDTA (pH 8.0), 20 mM sodium borate (pH 8.3), and 8.25% formaldehyde (Millipore, formaldehyde solution, 37%). The electrophoresis was carried out at 50 V using denaturing electrophoresis buffer containing 5% formaldehyde (Millipore; formaldehyde solution, 37%), 20 mM sodium borate (pH 8.3), and 0.5 mM EDTA (pH 8.0).

### cDNA preparation and qRT-PCR.

The cDNA synthesis was performed using 100 ng of RNA per gene, 500 nM specific reverse primer for the selected genes (Fig. S7B), 0.5 mM deoxynucleoside triphosphate (dNTP) mix, 0.8 U of RevertAid premium reverse transcriptase (Thermo Fisher Scientific), and 0.8 U of Ribolock in the presence of 1× RT buffer in RNase-free water. The denaturation was performed at 65°C for 5 min, annealing at 56°C for 30 min, and inactivation of the enzyme was carried out at 85°C for 10 min (Agilent Technologies; SureCycler 8000, PCR machine was used). Subsequently, the quantitative PCR was carried out using 10 μl of EvaGreen qPCR Mastermix-ROX (G-Biosciences), 500 nM (each) the gene-specific forward and reverse primer, and 2 μl of cDNA per well per reaction in a 96-well PCR plate. A CFX96 real-time PCR detection system (Bio-Rad) machine was used for qPCR. The values were analyzed using Bio-Rad CFX manager 3.1. The values obtained for each gene were normalized using 16S rRNA and corresponding gene in MLP (55). For all the respective gene, technical triplicates of the biological duplicates were made. Reactions were started with initial denaturation at 95°C for 5 min and amplification with 40 cycles of three-stage amplification (95°C for 10 sec, annealing for 20 sec at 55°C, and amplification at 72°C for 20 sec. The comparative Ct (ΔΔCt) method was used for the calculation of fold change in the expression levels of mRNA (56).

### Data availability.

All the data needed to evaluate the conclusions in the paper are given in the paper and/or in the supplemental materials. Additional data related to this paper may be requested from the corresponding author.
