# Supplementary figures and images for: Unique Mode of Cell Division by the Mycobacterial Genetic Resister Clones Emerging De Novo from the Antibiotic-Surviving Population
Source: mSphere. 2020 Nov 18;5(6):e00994-20. doi: 10.1128/mSphere.00994-20 (PMC7677009; doi:10.1128/mSphere.00994-20)

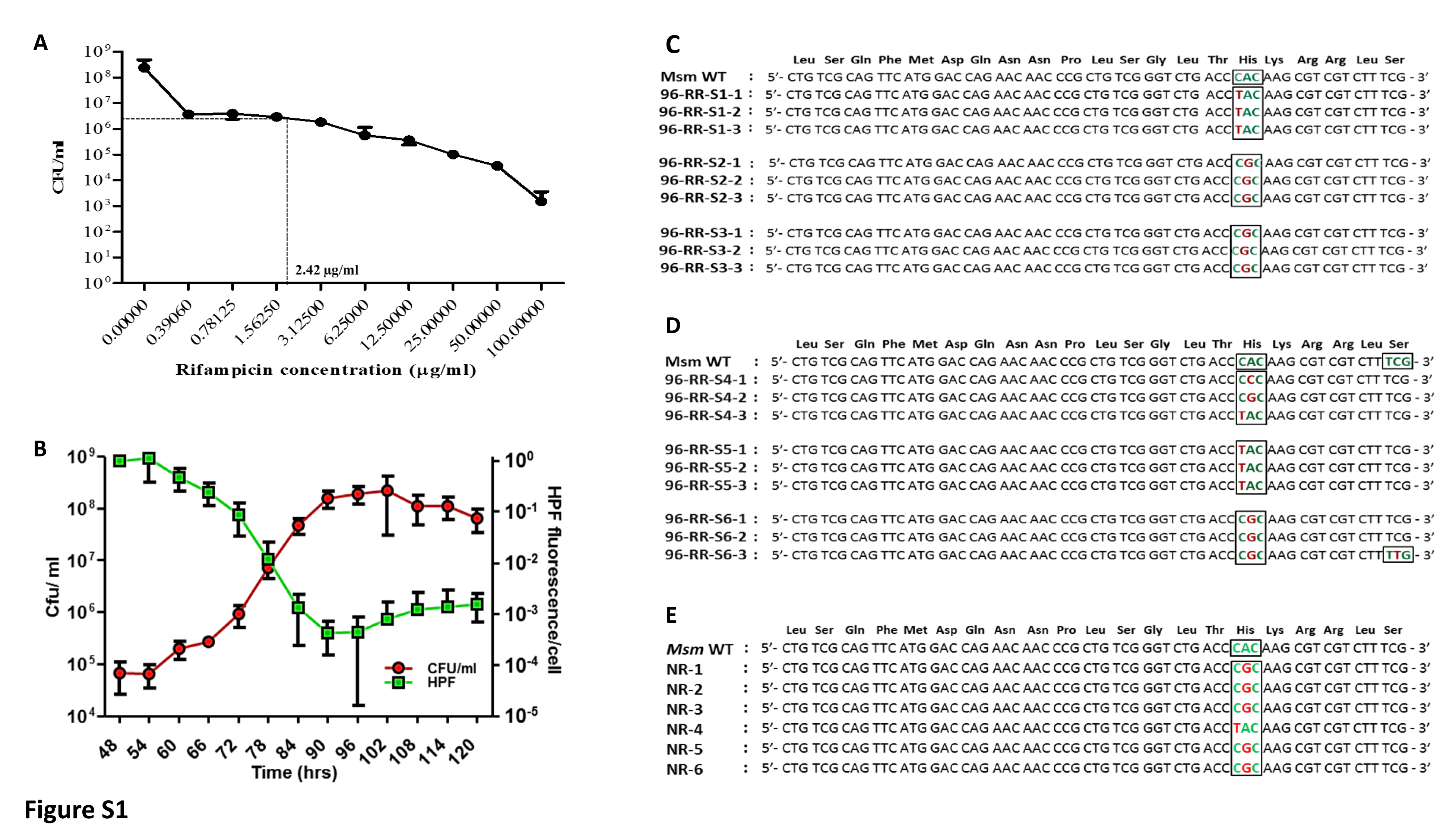

Supplement: FIG S1 [file mSphere.00994-20-sf001.tif]

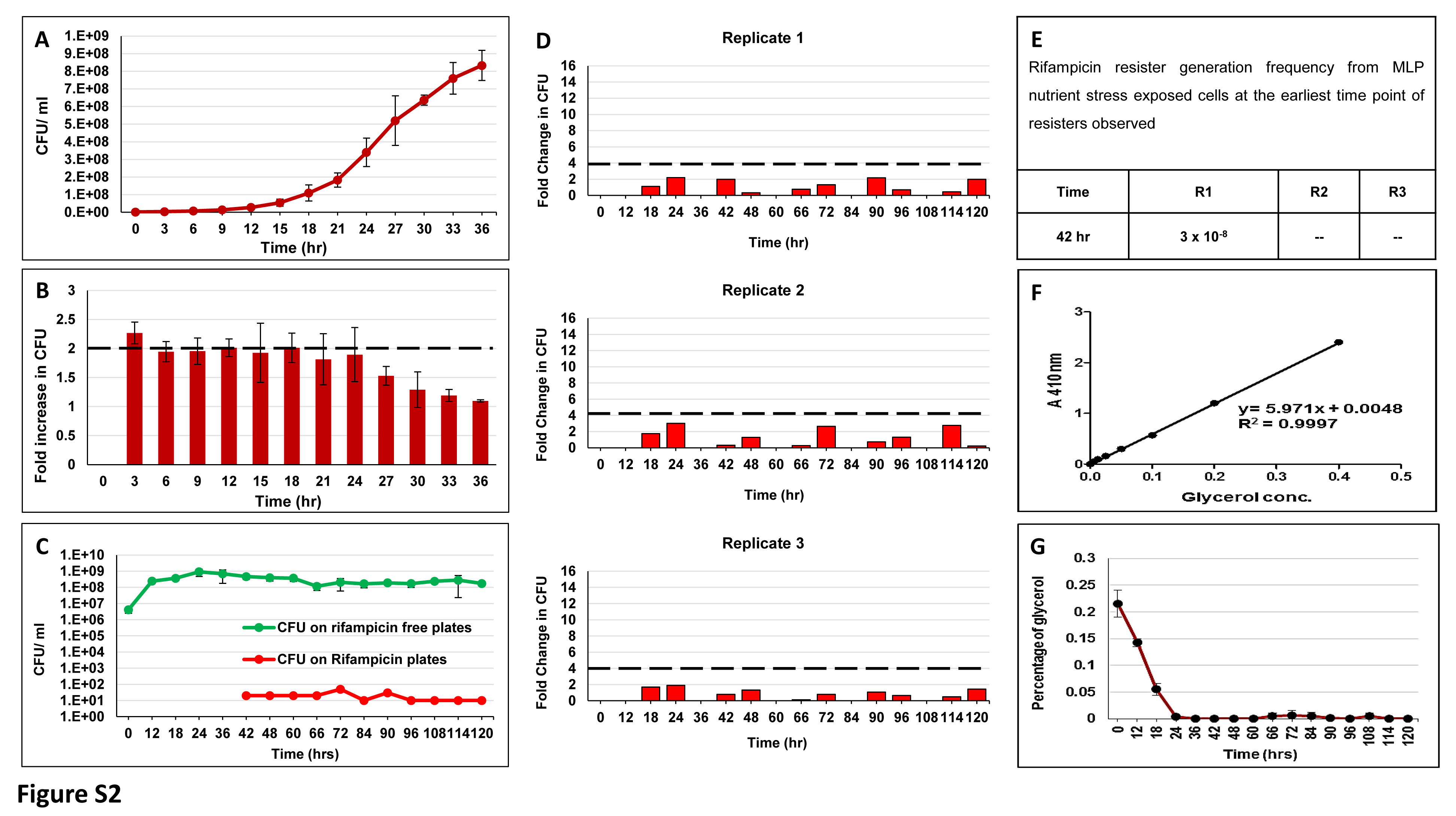

Supplement: FIG S2 [file mSphere.00994-20-sf002.tif]

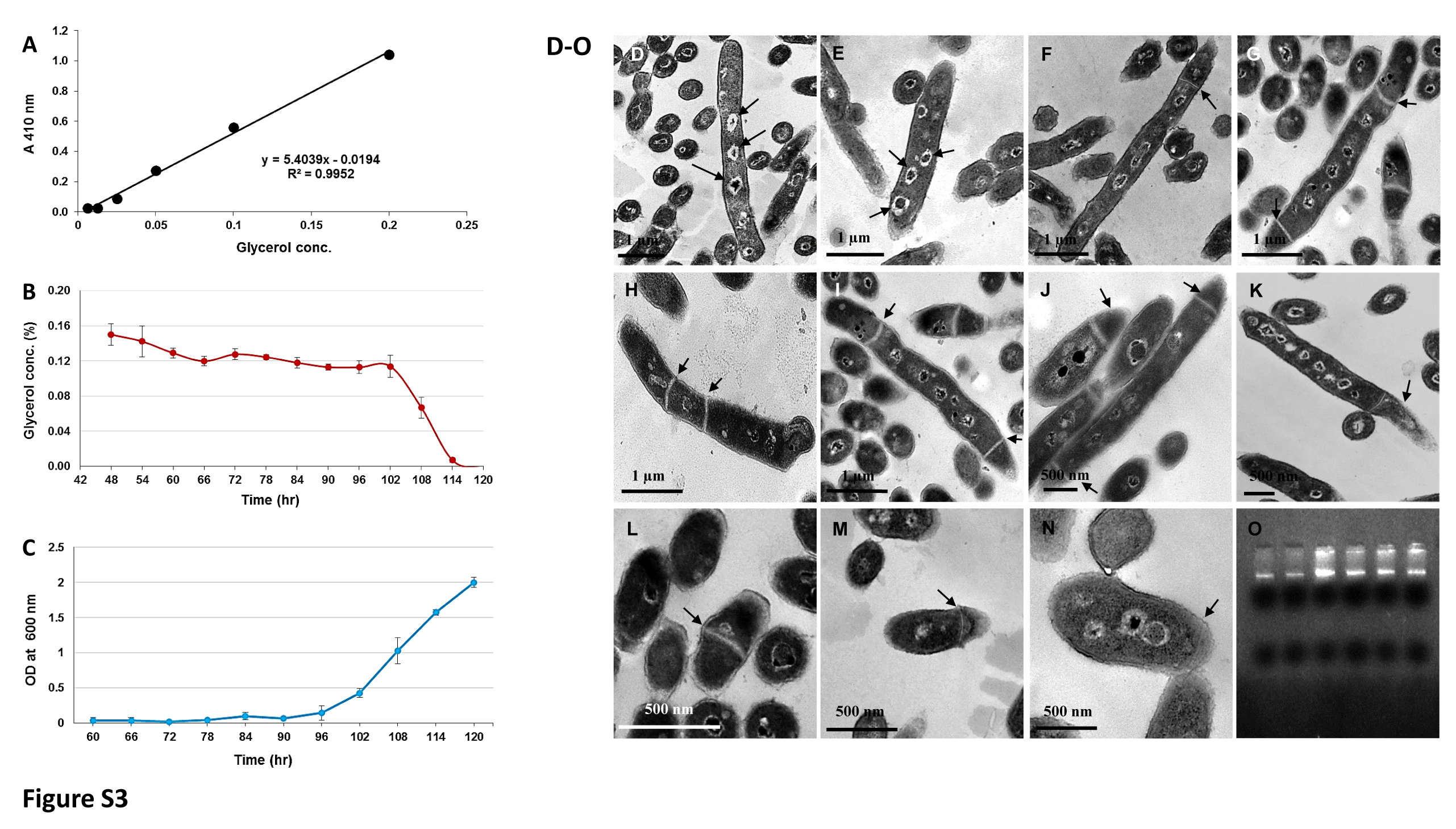

Supplement: FIG S3 [file mSphere.00994-20-sf003.tif]

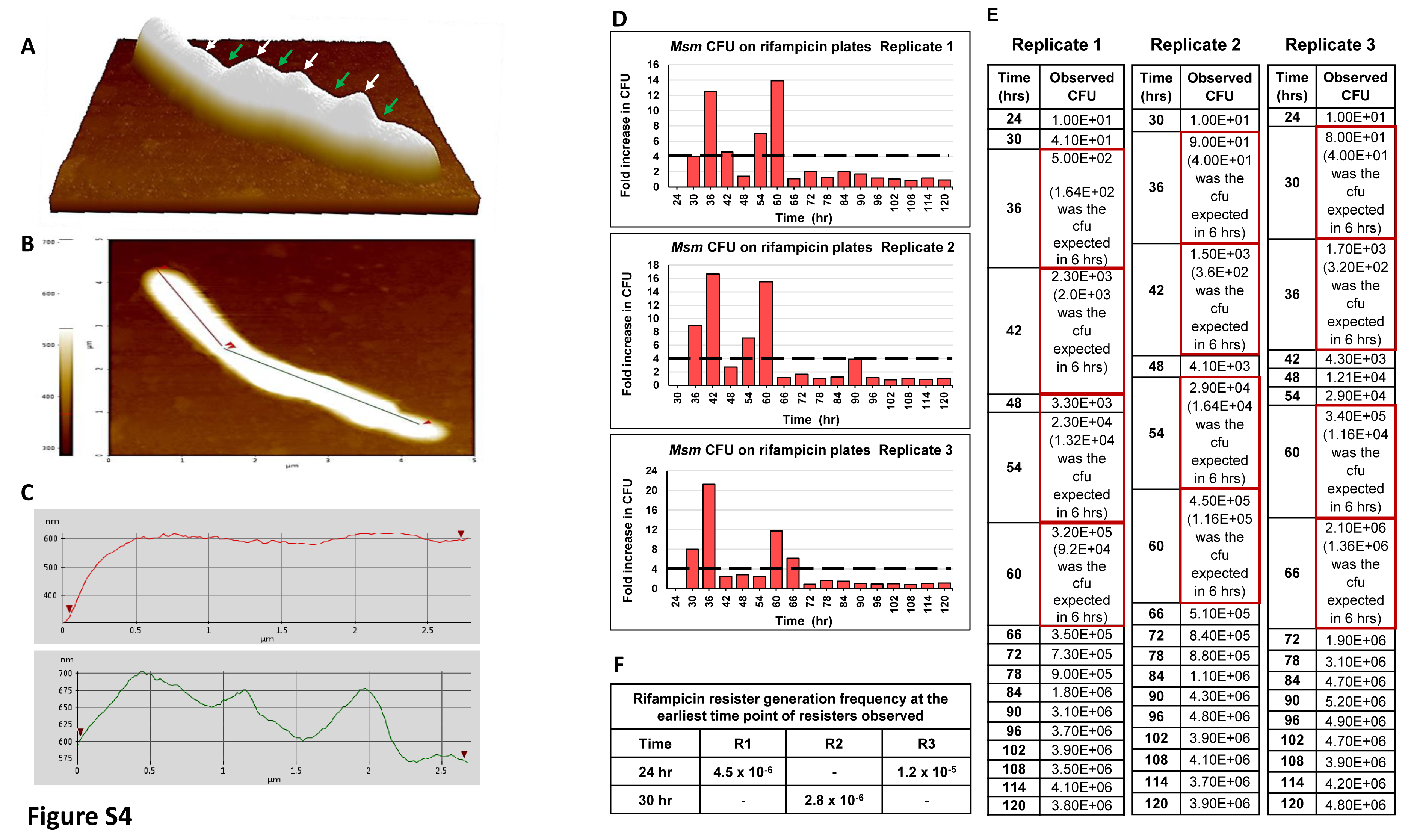

Supplement: FIG S4 [file mSphere.00994-20-sf004.tif]

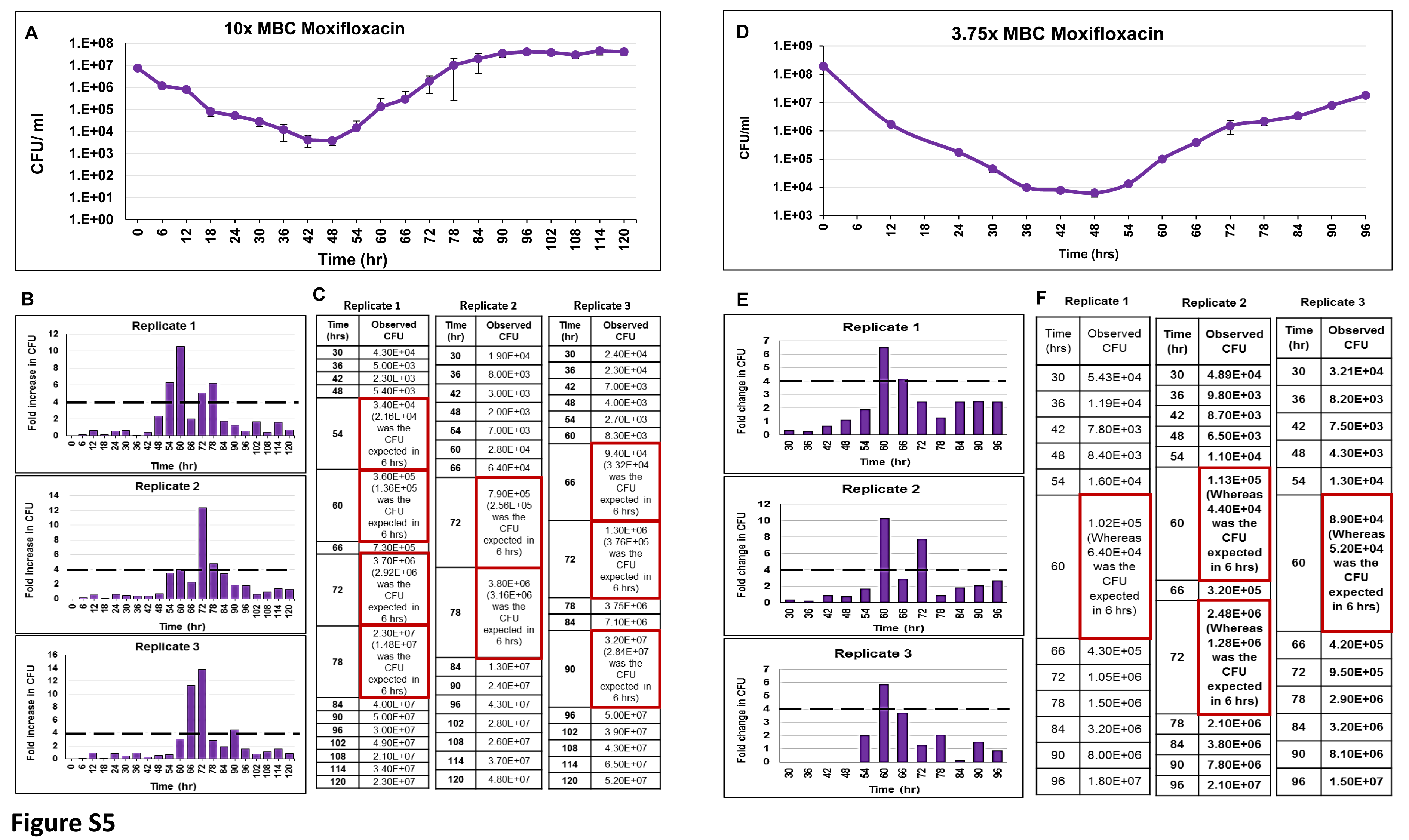

Supplement: FIG S5 [file mSphere.00994-20-sf005.tif]

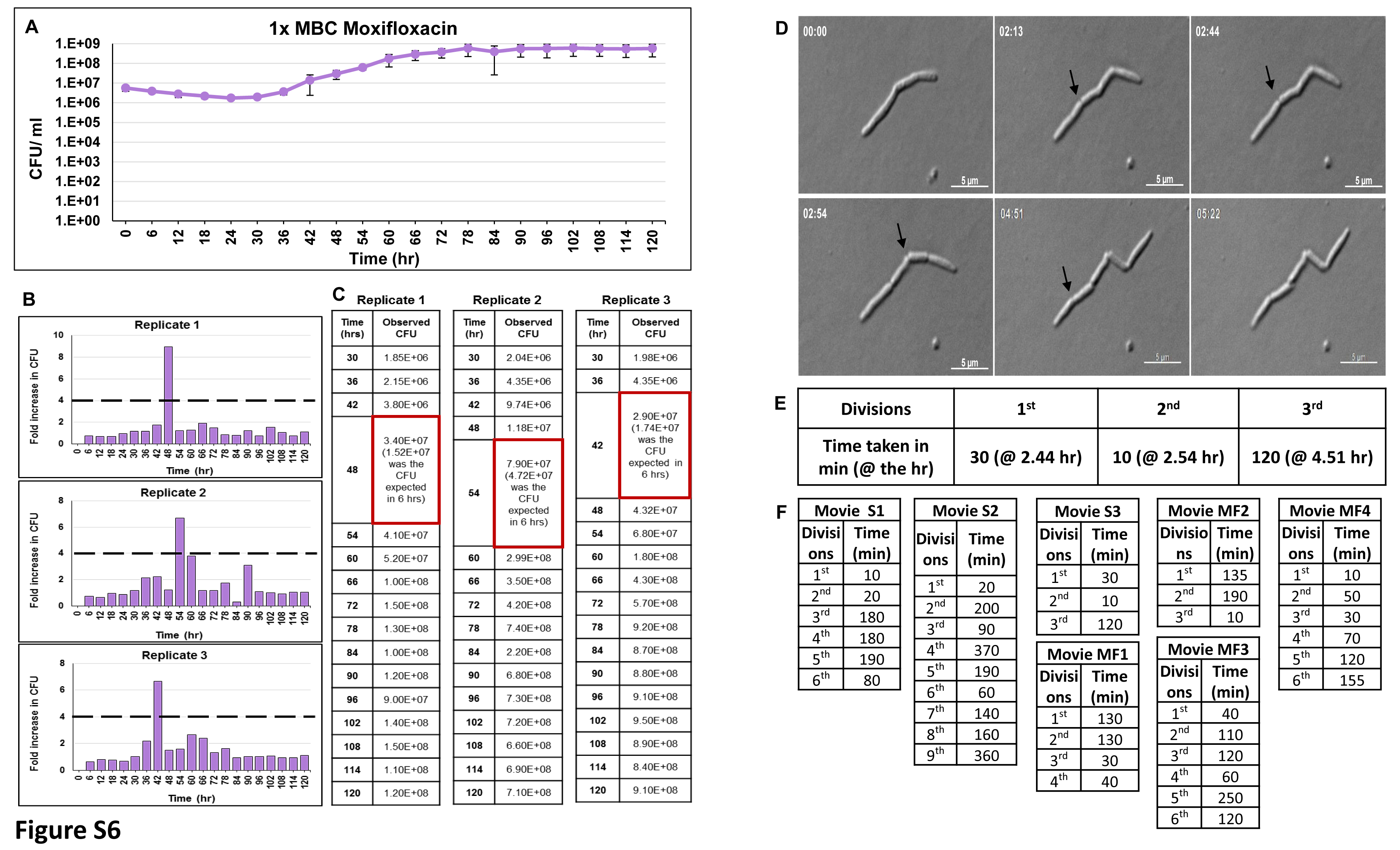

Supplement: FIG S6 [file mSphere.00994-20-sf006.tif]

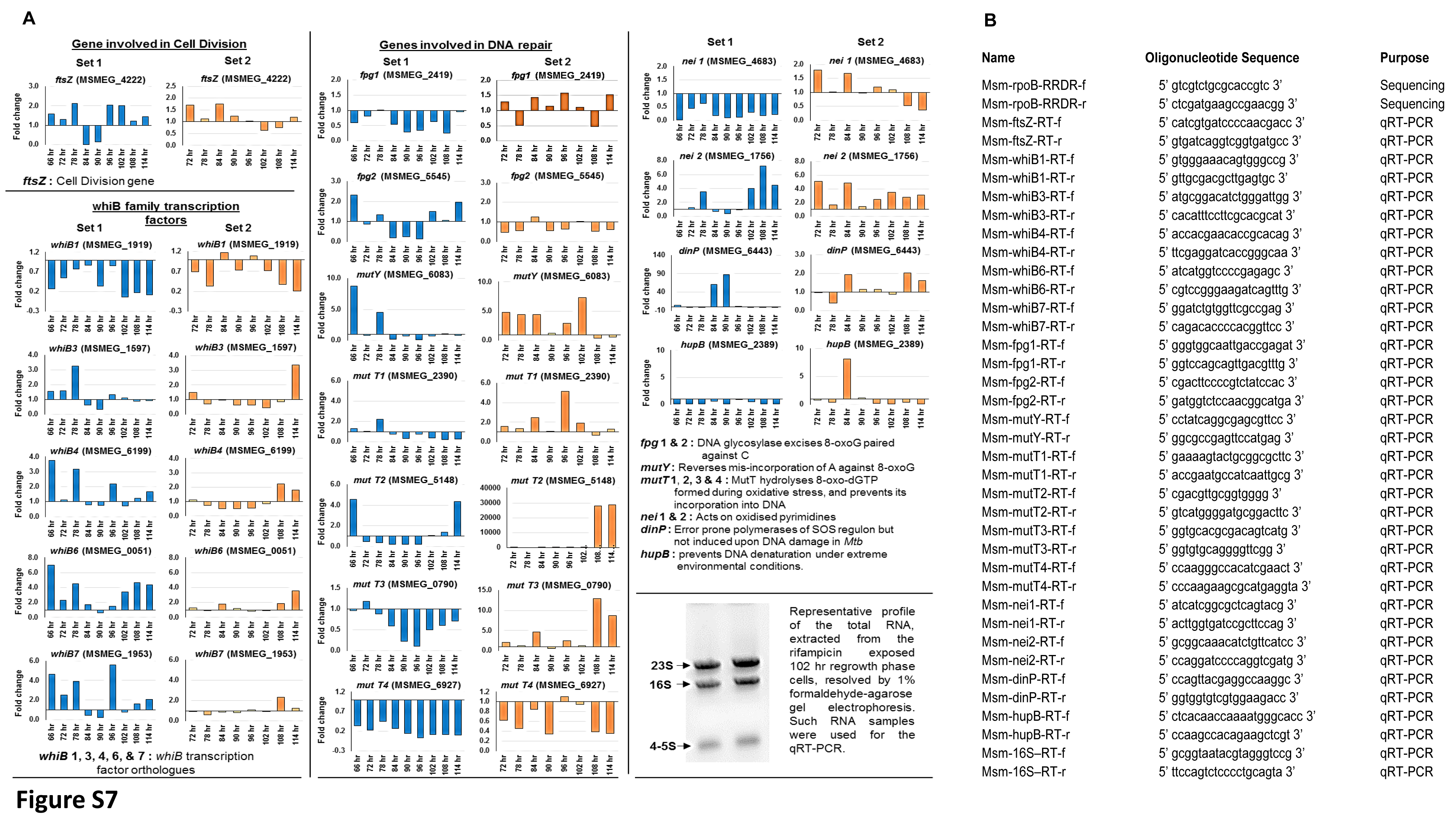

Supplement: FIG S7 [file mSphere.00994-20-sf007.tif]
